# Mapping molecular landmarks of human skeletal ontogeny and pluripotent stem cell-derived articular chondrocytes

**DOI:** 10.1038/s41467-018-05573-y

**Published:** 2018-09-07

**Authors:** Gabriel B. Ferguson, Ben Van Handel, Maxwell Bay, Petko Fiziev, Tonis Org, Siyoung Lee, Ruzanna Shkhyan, Nicholas W. Banks, Mila Scheinberg, Ling Wu, Biagio Saitta, Joseph Elphingstone, A. Noelle Larson, Scott M. Riester, April D. Pyle, Nicholas M. Bernthal, Hanna KA Mikkola, Jason Ernst, Andre J. van Wijnen, Michael Bonaguidi, Denis Evseenko

**Affiliations:** 1 Department of Orthopaedic Surgery, Keck School of Medicine of USC, University of Southern California (USC), Los Angeles, CA 90033 USA; 2 Department of Stem Cell Research and Regenerative Medicine, USC, Los Angeles, CA 90033 USA; 3 Bioinformatics Interdepartmental Program, UCLA, Los Angeles, CA 90095 USA; 4 Department of Biological Chemistry, UCLA, Los Angeles, CA 90095 USA; 5 Eli and Edythe Broad Center of Regenerative Medicine and Stem Cell Research at UCLA, Los Angeles, CA 90095 USA; 6 Department of Molecular, Cell and Developmental Biology, UCLA, Los Angeles, CA 90095 USA; 7 Institute of Molecular and Cell Biology, University of Tartu, Tartu, 51010 Estonia; 8 InVitro Cell Research, LLC, Cockeysville, MD 21030 USA; 9 Departments of Orthopedic Surgery & Biochemistry and Molecular Biology, Center of Regenerative Medicine, Mayo Clinic, Rochester, MN 55905 USA; 10 Department of Orthopaedic Surgery, David Geffen School of Medicine, UCLA, Los Angeles, CA 90095 USA; 11 Computer Science Department, University of California, Los Angeles, CA 90095 USA; 12 Jonsson Comprehensive Cancer Center, University of California, Los Angeles, CA 90095 USA; 13 Molecular Biology Institute, University of California, Los Angeles, CA 90095 USA

## Abstract

Tissue-specific gene expression defines cellular identity and function, but knowledge of early human development is limited, hampering application of cell-based therapies. Here we profiled 5 distinct cell types at a single fetal stage, as well as chondrocytes at 4 stages in vivo and 2 stages during in vitro differentiation. Network analysis delineated five tissue-specific gene modules; these modules and chromatin state analysis defined broad similarities in gene expression during cartilage specification and maturation in vitro and in vivo, including early expression and progressive silencing of muscle- and bone-specific genes. Finally, ontogenetic analysis of freshly isolated and pluripotent stem cell-derived articular chondrocytes identified that integrin alpha 4 defines 2 subsets of functionally and molecularly distinct chondrocytes characterized by their gene expression, osteochondral potential in vitro and proliferative signature in vivo. These analyses provide new insight into human musculoskeletal development and provide an essential comparative resource for disease modeling and regenerative medicine.

## Abstract (web summary)

Human development provides a roadmap for advancing pluripotent stem cell-based regenerative therapies. Here the authors mapped human skeletogenesis using RNA sequencing on 5 cell types from a single foetal stage as well as chondrocytes at 4 stages in vivo and 2 stages during in vitro differentiation.

## Introduction

Lineage specification and diversification are critical processes during development as cells with broad potential become restricted to specific lineages as they differentiate. This process has been best studied at the molecular level in model organisms, while comparatively little is known about human musculoskeletal development beyond anatomical characterization and analysis of core regulatory genes. The formation of the early limb bud is a complex case study in fate choice as lineage tracing experiments in mice have shown that Sox9 expression identifies a population of skeletogenic progenitors that can form cartilage, bone, ligament and tendon^1,2^. These fate decisions are dependent on local signaling cues and transcription factors including Runx2^3^, Osterix (Sp7)^4^ and Scleraxis (Scx)^5^. Osteoblastic progenitors segregate out of the Sox9^+^ population first, followed by tenocytes and ligamentocytes. Skeletal muscle, unlike limb cartilage, ligament, tendon and bone, is not derived from lateral plate mesoderm, but instead arises from paraxial mesoderm^6,7^. Muscle progenitor cells identified by Pax3/7^8,9^, MyoD1^10^ and Myf5^11^ delaminate from the dermomyotome^12^ and migrate into the limb bud^7^ where they proliferate and differentiate in coordination with the developing connective tissues. These studies have provided a strong mechanistic foundation of vertebrate skeletogenesis from which further analysis of human development may be performed.

Many of the molecular mechanisms that regulate development are highly conserved between vertebrates and humans, but there are also relevant differences between mice and humans that must be better understood for further advancement of regenerative medicine and cell-based therapies. Previous studies comparing human and mouse development in kidney^13^, liver^14^, lung^15^ and blood^16^ have all noted significant transcriptional and regulatory variance between the two species, coupled with high levels of conservation in tissue-specific gene networks. Given the significant disparities in growth plate development^17,18^, tissue thickness^19,20^, mechanical forces^21^ and potential for regeneration^22,23^ between mice and humans, we reasoned that a more comprehensive understanding of the underlying gene expression signatures that drive specification, diversification and function of musculoskeletal tissues during human ontogeny would provide insight into the molecular mechanisms of human development required for important therapeutic advances.

Here we implemented RNA sequencing to generate cell type-specific transcriptomes for chondrocytes, osteoblasts, myoblasts, tenocytes and ligamentocytes at 17 weeks post-conception (WPC) of human development. We then employed Weighted Gene Co-expression Network Analysis (WGCNA) to define tissue-specific gene modules that represent each cell type. We next used WGCNA to evaluate how gene expression changes throughout human ontogeny and implemented differential expression analysis to compare different stages of human and mouse chondrogenesis in vivo, while also drawing comparisons between human in vivo chondrogenesis and in vitro pluripotent stem cell (PSC) differentiation. These comparisons, in conjunction with the tissue-specific modules, revealed transcriptional plasticity that decreases as chondrocytes mature, as well as similarities and differences in gene expression during mouse and human chondrogenesis. These data were supported by analysis of chromatin states, which demonstrated that cartilage genes accumulate activating histone modifications with increased developmental age or time in culture, while genes associated with other lineages acquire a repressed state. Finally, this coordinated analysis allowed us to identify integrin alpha 4 (ITGA4) as a marker of distinct chondrocyte subsets within adult articular cartilage, as cells which co-express bone morphogenetic protein receptor 1b (BMPR1B) possess osteochondral potential in vitro, are enriched for the skeletal progenitor markers SOX9, GLI1 and RUNX2, and have a transcriptional program suggesting increased proliferative capacity in vivo. Taken together, these data sets provide a unique resource to understand human skeletogenesis and importantly may contribute to the development of future tissue engineering and regenerative medicine applications.

## Results

### Transcriptional profiling of fetal musculoskeletal lineages

**Fig. 1:**
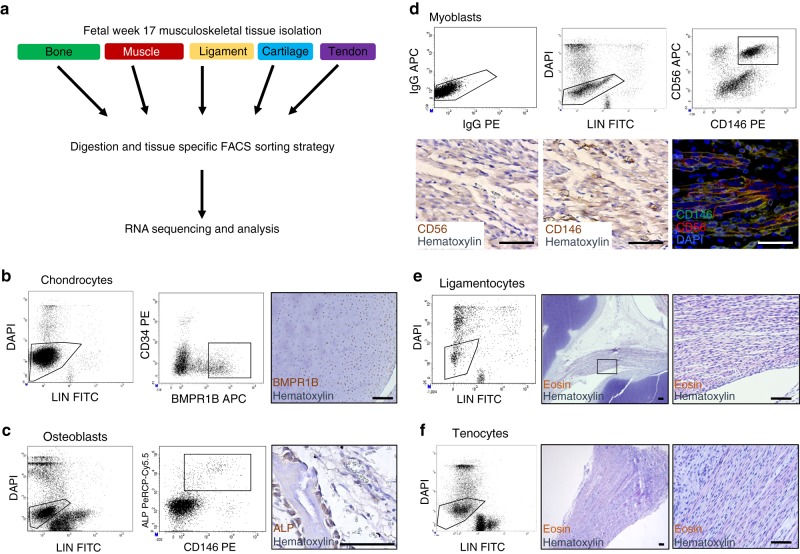
Identification and purification of five human fetal musculoskeletal lineages. **a** Strategy for assessing lineage diversification during human development. For all populations, cells were isolated as lineage (LIN) negative (CD235a^−^CD45^−^CD31^−^). **b** Chondrocytes were sorted as LIN^−^CD34^−^BMPR1B^+^ cells. BMPR1B positively labels the superficial zone of fetal articular cartilage. **c** Osteoblasts were sorted as LIN^−^ALP^+^ cells. ALP (alkaline phosphatase) positively labels endochondral osteoblasts in fetal trabecular bone. **d** Myoblasts were isolated based on LIN^−^CD56^+^CD146^+^ expression. Co-expression of CD56 (red) and CD146 (green) in fetal myoblasts was confirmed by immunofluorescence. **e** Ligamentocytes and **f** tenocytes were depleted of LIN^+^ cells following digestion of anterior/posterior cruciate ligament and Achilles tendons, respectively. *N* = 3–4; scale bars = 50 µm

To define unique and shared aspects of human musculoskeletal tissue specification and divergence, we employed a stringent cell isolation protocol based on flow cytometry followed by RNA sequencing. We first isolated cells from 17 WPC musculoskeletal tissues following overnight digestion at ambient oxygen: chondrocytes from the knee, myoblasts from the quadriceps, endosteal osteoblasts from the femur, and ligamentocytes and tenocytes from the anterior and posterior cruciate ligament and Achilles tendon, respectively (Fig. 1a). For all cell types, we gated out dead cells and the hematopoietic (CD45 and CD235a) and endothelial (CD31) lineages (LIN). Chondrocytes were sorted as BMPR1B^+^CD34^−^, as BMPR1B is highly enriched in the presumptive fetal articular cartilage and expressed throughout ontogeny^24^ (Fig. 1b, Supplementary Data 1) while CD34 identifies stromal cells. We isolated endosteal osteoblasts by selecting skeletal alkaline phosphatase^+^ (ALP^+^) cells^25^ (Fig. 1c). For muscle, we sorted CD146^+^ (MCAM)^26^ and CD56^+^ (NCAM)^27^ cells (Fig. 1d). For ligament and tendon, we sorted all LIN^−^ cells (Fig. 1e, f).

**Fig. 2:**
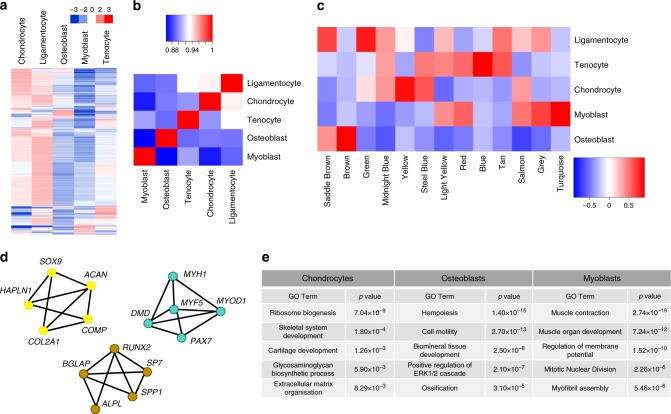
Identification of tissue-specific gene modules during human fetal musculoskeletogenesis. **a** Hierarchical clustering of the top 5000 expressed genes across all five cell types. **b** Spearman correlation analysis of the total transcriptomes of the same five tissues. **c** WGCNA delineated 13 modules based on patterns of gene co-expression in each tissue. Each column represents a unique gene module. Relative gene expression levels are indicated using the color scale. **d** String network demonstrating validity and connectivity of selected tissue-specific genes from the chondrocyte (Yellow), osteoblast (Brown) and myoblast (Turquoise) gene modules. **e** Selected Gene Ontology terms enriched in the chondrocyte, osteoblast and myoblast modules. *N* = 3–4

Hierarchical clustering of the 5000 most highly expressed genes across all tissues revealed large differences in gene expression across cell types, while a broad similarity in genes active in both chondrocytes and ligamentocytes was observed (Fig. 2a). This similarity was verified by a Spearman correlation matrix, which showed that gene expression in intra-articular ligamentocytes was most highly correlated with chondrocytes, followed by tenocytes (Fig. 2b). Lineage tracing performed in mice using a *Sox9*-Cre^ERT2^ allele demonstrated that cartilage, ligament and tendon can originate from mesenchymal progenitors identified by *Sox9* expression at E11.5-E13.5^1^. Our data suggest a similar phenomenon in human development and may provide insight into the timing and molecular basis for the diversification of these tissues.

### Generation of human tissue-specific gene modules by WGCNA

To identify genes uniquely enriched in each cell type, we utilized Weighted Gene Co-Expression Network Analysis (WGCNA)^28^. Genes with similar transcriptional dynamics across tissues were grouped into modules; each gene can only appear in a single module, and genes that did not fit the broader pattern of any module were excluded from the analysis. WGCNA of human fetal musculoskeletal tissues defined 13 distinct gene modules (Fig. 2c; Supplementary Data 2). Of note, each tissue was associated with a module whose expression was primarily enriched in it, *e*.*g*. the brown module represents osteoblasts (Fig. 2c). We verified the accuracy of these modules by examining the module assignments of well-defined tissue-specific genes, including key regulatory factors such as *SOX9*, *RUNX2* and *MYOD1* (Fig. 2d). We also identified multiple Gene Ontology (GO)^29,30^ terms relevant to their respective modules (Fig. 2e). To facilitate further characterization of novel tissue-specific genes enriched during musculoskeletal development, we generated a table for each module listing membrane-associated proteins and transcription factors (Supplementary Data 3–7). In addition, we included GO analysis of each module to provide a resource for identifying pathways influencing tissue diversification and maturation. We also employed OPPOSUM^31^ to identify transcription factor binding motifs enriched in the promoter regions of genes in each module (Supplementary Data 8), identifying both known and potentially novel transcription factors that may regulate each lineage. Finally, as an example of the multi-faceted value of this data set, Hicks et al.^32^ defined novel myogenic populations during human development and PSC differentiation that enrich for engraftable, muscle-forming cells. Together, this resource presents unique molecular insight into human musculoskeletal development and will support future research in developmental and regenerative biology.

### Transcriptional dynamics in human and mouse chondrogenesis

**Fig. 3:**
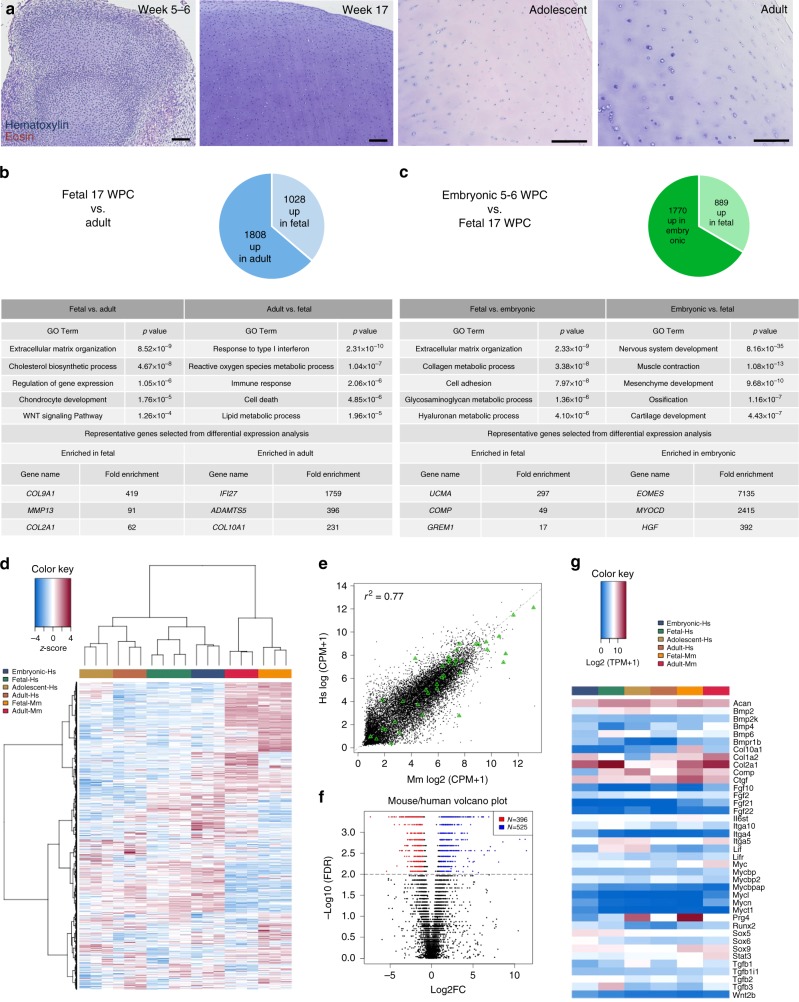
Transcriptional differences across human and mouse cartilage development. **a** Hematoxylin and Eosin (H&E) staining showing tissue architecture of chondrocytes at each stage profiled. **b** Selected Gene Ontology (GO) terms and genes enriched between fetal 17 WPC (weeks post conception; left) or adult (right) chondrocytes. **c** Selected GO terms and genes enriched between fetal 17 WPC (left) or embryonic 5–6 WPC (right) chondrocytes. **d** Hierarchical clustering of 15,000 genes expressed across 4 stages of human ontogeny and 2 stages of mouse development. **e** Mean enrichment plot between mouse and human samples. Green triangles represent selected chondrogenic genes. **f** Volcano plot showing differentially expressed genes between mouse and human. **g** Heat map (logarithmic expression) of chondrogenic genes shown in mean enrichment plot across all human and mouse samples. Hs: human, Mm: mouse, FC: fold change. *N* = 3–4; scale bars = 50 μm

To better understand the molecular basis underlying human cartilage specification and development, we performed a longitudinal transcriptional analysis of chondrocytes across 4 different stages of human ontogeny, including pre-chondrocytes isolated from limb bud condensations at embryonic 5–6 WPC, BMPR1B^+^ presumptive articular chondrocytes^24^ from 17 WPC and healthy juvenile and adult BMPR1B^+^ articular chondrocytes^24^ (Fig. 3a). Principal Component Analysis (PCA) (Supplementary Figure 1a) and hierarchical clustering of 15,000 genes showed clear differences across different stages of human ontogeny and also considerable areas of conservation, especially between the embryonic and fetal developmental stages (Supplementary Figure 1b). To better determine which genes were specifically enriched at each stage, we used WGCNA to define 9 distinct gene modules (Supplementary Figure 1c, Supplementary Data 9). Similar to our observations from hierarchical clustering, there are several modules shared between embryonic and fetal stages of development while single modules representative of embryonic, fetal, adolescent and adult stages also segregate in the analysis. Differential expression analysis revealed that the genes upregulated in the adolescent stage compared to fetal (Supplementary Data 10–11) have significant similarity (*p* = 0; hypergeometric test) to those genes enriched in adult cartilage compared to fetal, suggesting adolescent chondrocytes are strongly similar to adult chondrocytes (Supplementary Figure 1d, e). Despite these large similarities, the genes differentially expressed between adolescent and adult chondrocytes (Supplementary Figure 1d, Supplementary Data 12–13) indicated that adolescent cells are more proliferative while adult cells have increased levels of epigenetic and post-transcriptional regulation (Supplementary Figure 1f).

We next implemented differential expression coupled with GO and OPOSSUM analyses to identify genes, pathways and motifs enriched at each stage of human chondrogenesis. Comparison of fetal to adult articular chondrocytes (Fig. 3b, Supplementary Data 14) revealed that fetal chondrocytes evidenced active matrix deposition, a hallmark of the chondrogenic program. Conversely, adult chondrocytes were enriched for genes associated with metabolism, cell death and interferon signaling, suggesting that older articular chondrocytes have comparatively reduced matrix deposition. Comparison of fetal chondrocytes with embryonic pre-chondrocytes (Fig. 3c, Supplementary Data 15) identified a similar enrichment of chondrogenic properties in 17 WPC cells, highlighted by GO terms such as extracellular matrix (ECM) organization, cell adhesion and collagen metabolism. In contrast, there remained substantial transcriptional plasticity at the embryonic stage, evidenced by higher levels of transcripts associated with muscle, neural and bone lineages. This was corroborated by promoter motif analysis of embryonic-enriched genes, which suggested potential regulation by both neural (SOX2) and chondrogenic (SOX5/9) SOX family members, as well as the cardiac transcription factor NKX2.5. At the fetal stage, motifs for RELA and NF-κB were enriched, along with those bound by ZFX, MZF family members and SP1; genes upregulated in adult chondrocytes were also enriched for these motifs (Supplementary Data 16). Notably, binding sites for these three transcription factors were also enriched in the chondrocyte module (Supplementary Data 8). SP1 has been described to influence collagen expression^33^, while ZFX and MZF family members have not been associated with chondrocyte biology. Overall, these findings strongly suggest that embryonic pre-chondrocytes, although committed to the chondrogenic fate^24^, are still in the process of repressing genes associated with other mesodermal fates in a progressive silencing during development and maturation.

Our initial analysis of fetal chondrocytes compared to other musculoskeletal tissues revealed that many genes identified as important chondrogenic regulators through mouse studies were enriched in developing human cartilage, indicating there is a strong conservation of core regulatory components. To better evaluate our analysis of human chondrogenesis, we next compared these two species at the transcriptomic level. We isolated chondrocytes from E16.5 mouse embryos, a pre-natal stage where the articular layer is populated with permanent, resident articular chondrocytes^34^, and also from post-natal 2 month old adult animals. We then sorted LIN^−^ALP^−^BMPR1B^+^ cells for sequencing. Hierarchical clustering (Fig. 3d) and PCA (Supplementary Figure 2a) of all mouse and human samples demonstrated substantial transcriptomic differences between species. Human vs. mouse mean enrichment (Fig. 3e) and volcano plots (Fig. 3f) assessed as an aggregate of all stages demonstrate a normal distribution of gene expression and differentially expressed genes between the two species. Closer inspection of selected core chondrogenic genes (green triangles, Fig. 3e–g) revealed a high degree of conservation among these genes, with only one gene (*Col10a1*) > 1.5 standard deviations different than the mean (Wilcoxian signed rank test), suggesting the differences observed between species were unrelated to the core chondrogenic program. Differential expression analysis of genes upregulated in mouse chondrocytes revealed enrichment for WNT, AKT and MAPK signaling as well as cell cycle-related genes, suggesting mouse cells may proliferate more than human chondrocytes (Supplementary Figure 2b, Supplementary Data 17). Conversely, in human cells there was an upregulation of genes associated with oxidative metabolism and ribosome biogenesis (Supplementary Figure 2b, Supplementary Data 18). These findings support strong conservation of the core components of the chondrogenic program while differences in signaling and proliferation may underlie the disparity in regenerative potential between mouse and human articular cartilage.

### Transcriptional analysis of PSC-derived cartilage

**Fig. 4:**
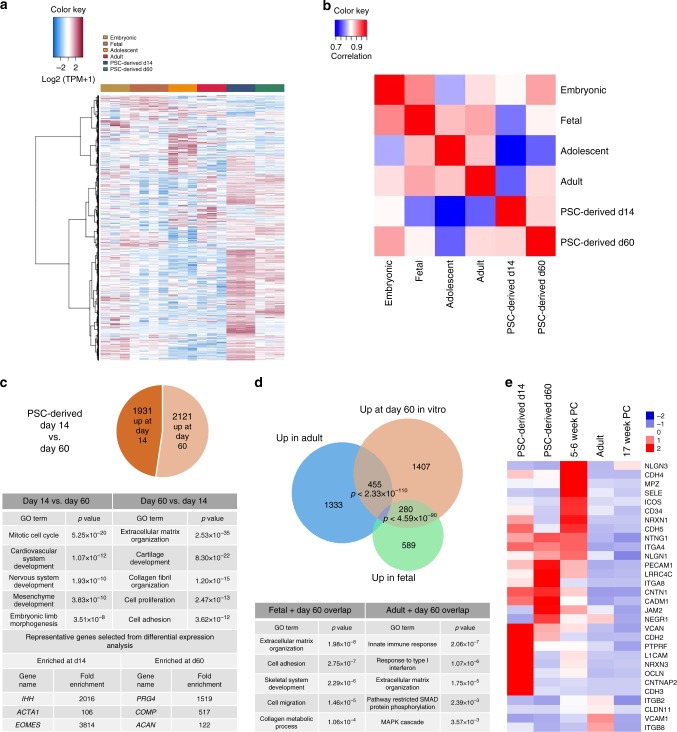
Transcriptional comparison of PSC-derived chondrocytes and in vivo human cartilage development **a** Hierarchical clustering of 15,000 genes expressed across all 4 stages of human ontogeny and 2 stages of PSC-derived chondrocytes. **b** Spearman correlation matrix of the relatedness of the samples. **c** Selected GO terms and genes enriched between d14 (left) and d60 (right) pluripotent stem cell-derived chondrocytes. **d** Genes enriched in d60 PSC-derived chondrocytes significantly overlap with those enriched in adult (adult vs. fetal) and fetal (fetal vs. embryonic) cells. Selected GO terms from these overlaps are shown. **e** Relative gene expression values at different stages of chondrogenesis and PSC-derived cartilage of cell adhesion molecules (CAMs) enriched in d14 vs. d60 chondrocytes. *N* = 3–4

We have previously demonstrated that lineage-committed chondrocytes are formed fourteen days after mesodermal induction of PSCs^24^ in vitro, and to better assess how well this process reflected human development we profiled the transcriptomes of these cells compared to cells allowed to mature for 60 days in culture. PCA (Supplementary Figure 3a), hierarchical clustering (Fig. 4a) and Spearman correlation analysis (Fig. 4b) of both in vitro stages compared to chondrocytes isolated during human ontogeny suggested that although d14 and d60 cells are most similar to one another, d60 cells are substantially more similar to in vivo-derived cells (Fig. 4a, b). This was further demonstrated by comparison of the transcriptomes of d14 and d60 cells (Fig. 4c; Supplementary Data 19), which indicated that in vitro maturation is broadly similar to cartilage specification in vivo, as GO categories enriched at the d60 stage strongly overlapped with those enriched in fetal vs. embryonic chondrocytes. There was also a strong overlap of genes enriched at day 60 with genes enriched in adult chondrocytes (Fig. 4d). Finally, closer inspection of cartilage-associated genes demonstrated increased maturation from d14 to d60 and close resemblance to in vivo chondrogenesis (Supplementary Figure 3b). These findings suggest that extended culture of PSC-derived chondrocytes generates a highly chondrogenic population of cells that may represent a developmental stage between fetal and adult chondrocytes.

### ITGA4 marks unique populations in adult articular cartilage

We next focused our analysis on identifying candidate surface markers of more primitive human chondrocytes. Our previous work^24^ identified BMPR1B-expressing chondrocytes as cells localized to the superficial and transitional zones of articular cartilage, known to contain cells with progenitor-like properties and enriched for the chondrogenic transcription factor *SOX9*^35^. Here we aimed to identify additional surface markers that could yield further insight in human articular cartilage heterogeneity. Specifically, we focused on cell adhesion molecules (CAMs) that were enriched in both PSC-derived populations and embryonic chondrocytes vs. fetal and adult cells (Fig. 4e). Of the 30 CAMs enriched in embryonic vs. fetal chondrocytes, only one of these genes was also present in the embryonic module identified by WGCNA (Supplementary Figure 1c, Supplementary Data 9): *ITGA4*. We confirmed expression of ITGA4 during human cartilage development by immunohistochemistry, and observed a strong presence in limb bud condensations that became progressively rarer over time, consistent with our expression analysis (Supplementary Figures 4a–i).

ITGA4 is a member of the integrin family of transmembrane receptors. Like other integrins, ITGA4 forms heterodimers to signal, partnering with integrins beta 1 and beta 7^36,37^. Upon heterodimerization, ITGA4 has been shown to be a receptor for fibronectin^38^; through this interaction, ITGA4 is thought to regulate cellular processes such as migration and motility^39^. Moreover, past research has identified a distinct population of more primitive cells capable of proliferation in adult cartilage based on enhanced binding to fibronectin^35^. On the basis of these data, and the expression profile of *ITGA4* at different stages of human chondrocyte ontogeny, we hypothesized it may distinguish specific populations in adult cartilage.

**Fig. 5:**
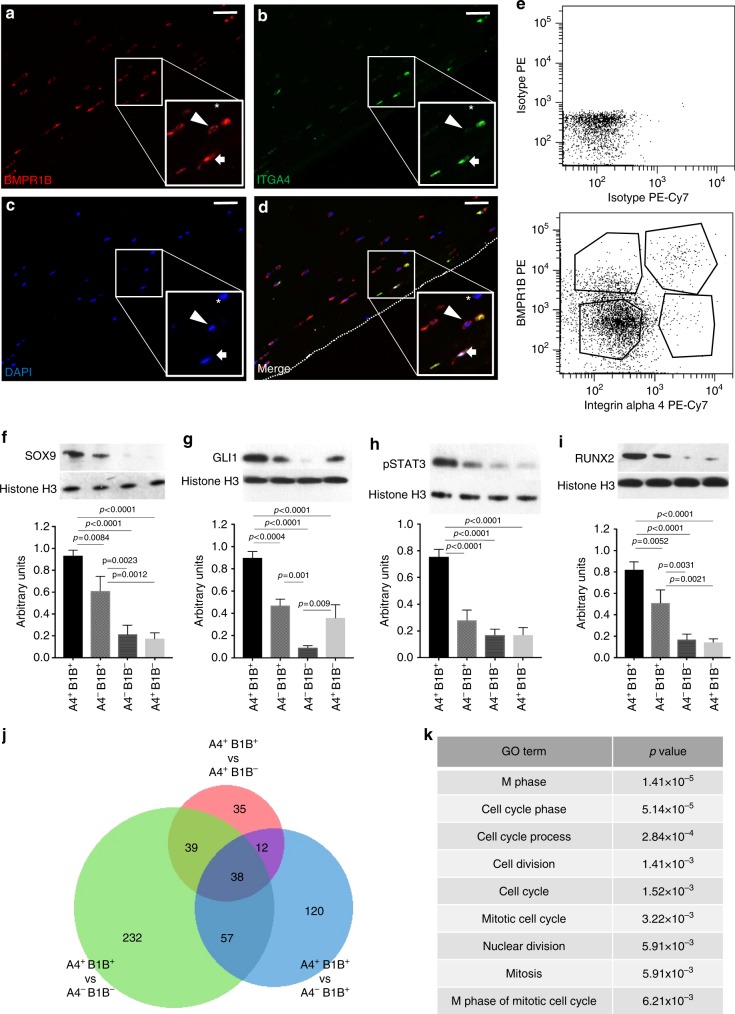
ITGA4^+^ defines molecularly distinct subsets of articular cartilage that are enriched for osteochondral proteins. **a** BMPR1B (red) identifies many cells of the superficial layer of adult human articular cartilage. **b** ITGA4 (green) labels rare cells in the most superficial region of adult human articular cartilage. **c** DAPI (blue) labels nuclei. **d** Merge channels. Arrow labels a BMPR1B^+^ITGA4^+^ cell, arrowhead labels a BMPR1B^+^ITGA4^−^cell and asterisk labels a BMPR1B^−^ITGA4^−^cell. Dotted line indicates articular surface. **e** Flow cytometry of adult pig chondrocytes identified four populations of cells based on expression of BMPR1B and ITGA4. **f**–**i** Western Blot analysis and quantification of protein expression in adult pig chondrocytes sorted on expression of BMPR1B (B1B) and ITGA4 (A4). SOX9 (f), GLI1 (**g**), pSTAT3 (**h**), and RUNX2 (**i**) are all significantly enriched in BMPR1B^+^ITGA4^+^ cells compared to other sorted populations as calculated by one-way ANOVA followed by Tukey’s multiple comparison test. **j** The four populations of sorted pig chondrocytes were sequenced and differential expression analysis was performed in pairwise fashion. 38 genes are uniquely enriched in BMPR1B^+^ITGA4^+^ cells compared to all other population. **k** GO analysis of these 38 genes. *N* = 3; scale bars = 50 μm

Immunohistochemical analysis of human adult cartilage samples taken from the tibial plateau revealed ITGA4 is expressed by a small subset of cells in the superficial layer, while the expression of BMPR1B was broader, enriched in both the superficial and proteoglycan-rich transitional zones (Supplementary Figures 4j–n). Immunofluorescent staining for both proteins demonstrated small subpopulations of superficial ITGA4^+^BMPR1B^+^ and ITGA4^+^BMPR1B^−^cells, along with more frequent ITGA4^−^BMPR1B^+^ and ITGA4^−^BMPR1B^−^cells (Fig. 5a–d). Most chondrocytes, especially those in the deeper zones, did not express either protein.

After confirming similar populations in both pig and mouse articular cartilage (Supplementary Figure 5a, b), we sorted four populations defined by the presence/absence of ITGA4 and BMPR1B from adult pig cartilage (Fig. 5e) and evaluated protein expression associated with more primitive fetal chondrocytes and skeletal progenitors (Fig. 5f–i). Our analysis demonstrated that BMPR1B^+^ITGA4^+^ chondrocytes are enriched for GLI1^40^, SOX9^2^, RUNX2^3^ and pSTAT3^41^, suggesting that they may represent a more plastic sub-population of chondrocytes within articular cartilage. We next performed RNA-seq on each chondrocyte population; in parallel, we sequenced multipotent CD45^−^CD31^−^CD146^+^ synovial pericytes^42^. Pairwise differential expression analysis revealed that each of the four chondrocyte populations were highly different from synovial pericytes (Supplementary Figure 5c, Supplementary Data 20–23) but relatively similar to one another (Supplementary Figure 5d, Supplementary Data 24–29). Genes most enriched in ITGA4^+^BMPR1B^+^ cells compared to CD146^+^ pericytes included *COL2A1, ACAN, COMP, SOX9* and other cartilage-specific genes that fall in the chondrocyte gene module (Supplementary Figure 5e), representing a highly significant overlap. Conversely, there was little variation in cartilage-specific genes between ITGA4^+^BMPR1B^+^ and ITGA4^−^BMPR1B^+^ cells (Supplementary Figure 5e), although we did note that the important proteoglycan PRG4/lubricin, a marker of the most superficial chondrocytes, was enriched in both ITGA4^+^ populations; we confirmed this finding by qPCR (Supplementary Figure 5f). We next asked if there was a transcriptional signature associated with the rarest ITGA4^+^BMPR1B^+^ chondrocytes. Overlap of genes enriched in ITGA4^+^BMPR1B^+^ compared to the three other populations revealed a small cohort of 38 genes (Fig. 5j) that is uniquely enriched in ITGA4^+^BMPR1B^+^ vs. all 3 populations. Interestingly, GO analysis on these 38 genes revealed enrichment for terms associated with the cell cycle, mitosis and proliferation (Fig. 5k).

To test for functional differences between these four chondrocyte populations residing within adult articular cartilage, we sorted cells from pig knee joints and cultured them for one passage; morphological differences among the populations were immediately apparent. While the double negative and ITGA4^−^BMPR1B^+^ cells displayed a typical chondrocyte morphology, the ITGA4^+^ populations evidenced a more fibroblast-like morphology with multiple long projections (Supplementary Figure 6a). Given this observation combined with the enrichment of proteins associated with skeletal progenitors, we assessed the osteogenic and chondrogenic differentiation potential of these populations in vitro (Supplementary Figure 6b–d). As expected, all populations were chondrogenic, with the strongest matrix generating potential evident in the ITGA4^−^BMPR1B^+^ cells which were mainly localized in the transitional zone. Notably, ITGA4^+^BMPR1B^+^ cells were robustly osteogenic, while the other populations evidenced little capacity to form bone, in agreement with the robust levels of RUNX2 in these cells (Fig. 5i). ITGA4^−^BMPR1B^−^cells demonstrated minimal or no osteogenic and matrix-producing potential, consistent with their localization in the deep zone of articular cartilage and likely reflecting their terminal differentiation status. Together, these findings demonstrate that ITGA4 expression defines at least two subsets within articular cartilage: robustly chondrogenic ITGA4^−^BMPR1B^+^ cells and ITGA4^+^BMPR1B^+^ cells that are enriched for osteochondral progenitor markers and have a proliferative gene expression signature in vivo and osteochondral differentiation capacity in vitro.

### Epigenetic regulation of in vivo and in vitro chondrogenesis

It has been previously demonstrated that the transcription factor Sox9 is master regulator of a chondrogenic network of genes in mice. The presence of Sox9 at promoter and enhancer elements strongly correlated with active gene expression and positive epigenetic regulatory marks^43^. Having defined the transcriptional program which distinguishes cartilage from other musculoskeletal tissues at 17 WPC in humans, we hypothesized that these genes are also highly regulated at the epigenetic level. Accordingly, we performed chromatin immunoprecipitation followed by DNA-sequencing (ChIP-Seq) across different chondrocyte populations, including fetal chondrocytes, adult articular chondrocytes and PSC-derived chondrocytes at d14 and d60. To assess the epigenetic landscape, we immunoprecipitated DNA/protein complexes with antibodies against H3K27ac, H3K27me3, H3K4me1 and H3K4me3. Together, these four histone modifications can be used to identify “chromatin states” at individual genomic loci or to find trends across the entire genome^44^.

**Fig. 6:**
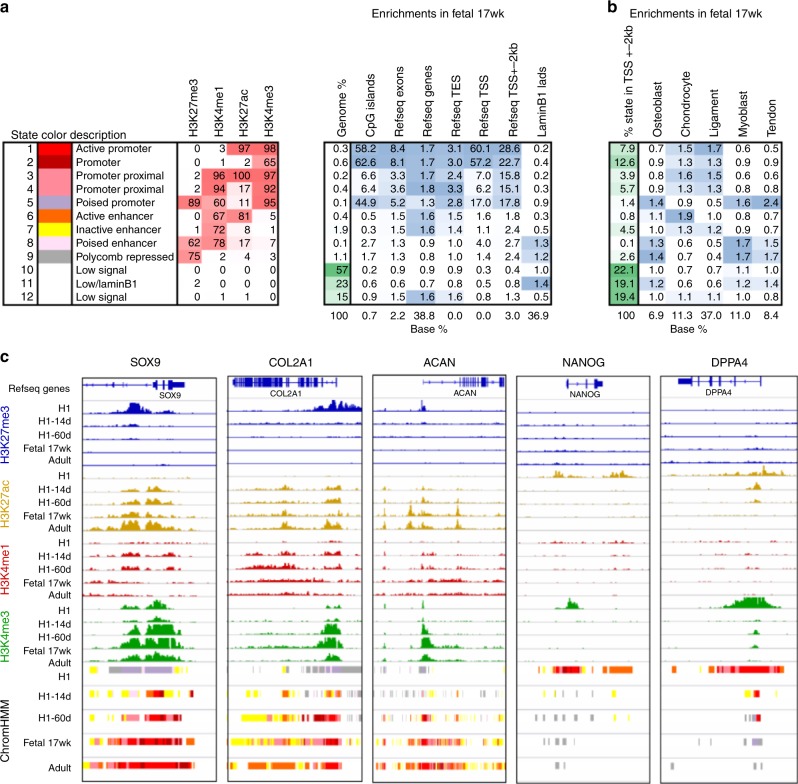
Human fetal chondrocyte identity is regulated at the epigenetic level. **a** Left panel: Chromatin state definitions (emission probabilities) for the 12 state ChromHMM model. Rows correspond to chromatin states. Columns correspond to chromatin state number, color code, candidate description and histone marks. Numbers in the histone mark columns show the frequency of occurrence of each mark in each chromatin state on the scale from 0 (white) to 100 (red). Right panel: Enrichment scores for chromatin states in genomic features. The first column shows the genome-wide percentage of occupancy for each state on the scale from 0 (white) to 100 (green). Subsequent columns show enrichments for CpG islands, Refseq annotated exons, genes, transcription ends sites (TES), transcription start sites (TSS), TSS + −2 kb regions and laminB1 domains. Each column is colored from 0 (white) to its maximum value (blue). The bottom row shows the baseline percentage of genomic territory occupied by each feature. **b** Enrichments of chromatin states from Fetal 17-weeks in TSS proximal regions (defined as TSS + −2 kb) of genes from different gene expression modules identified by WGCNA. The first column shows the baseline percentage occupied by each chromatin state in all annotated TSS + −2 kb regions on the scale from 0 (white) to 100 (green). Subsequent columns show the enrichments for each state in each expression module based on the fraction occupied by that state in the TSS proximal regions of all genes. Each column is colored from 0 (white) to its maximum value (blue). The bottom row shows the baseline fraction of each module out of all genes. Promoters of cartilage and ligament genes are enriched for active promoter states, whereas bone, muscle and tendon genes are enriching for Polycomb-repressed, poised and low signal states. **c** Chromatin data from all four cell types interrogated in this study and from H1-hESC at specific genes involved in either cartilage development (SOX9, COL2 and ACAN) or pluripotency (NANOG and DPPA4). ChromHMM tracks are colored according to the color codes in **a**. *N* = 3–4

We utilized ChromHMM^44^ to generate 12 chromatin states for all cell types (Supplementary Data 30–33). These states include active promoter (AP; enriched for H3K4me3 and H3K27ac), promoter proximal (PP; enriched for H3K4me1, H3K4me3 and H3K27ac), poised enhancer (H3K4me1 and H3K27me3) and polycomb repressed (PR; high H3K27me3) regions, among others (Fig. 6a). We first examined these chromatin states at a global level across the lineage-specific modules in fetal chondrocytes. Enrichment analysis of chromatin state distribution in promoter proximal regions shows an extensive level of epigenetic regulation with significant alignment with our expression data (Fig. 6b, Supplementary Data 30). In fetal chondrocytes, both AP and PP states are enriched amongst genes in the chondrocyte and ligament modules, while levels of the PR state are lower compared to all genes. Conversely, the PR state is enriched in genomic regions associated with genes in the osteoblast, myoblast and tendon modules, while the active promoter states are reduced across these three modules (Fig. 6b and Supplementary Figures 7b–d). This general pattern was consistent across cartilage populations (Supplementary Figure 7a). Of note, at 17 WPC the PR state is not enriched in the ligament module, which likely reflects the close transcriptional similarity between these two lineages at this developmental stage. Furthermore, closer inspection of loci that are known to be highly expressed in cartilage and are present in the chondrocyte module demonstrates very specific enrichment of “active” histone modifications, H3K4me3 and H3K27ac, while the repressive H3K27me3 is largely absent (Fig. 6c). Conversely, the pluripotency genes *NANOG* and *DPPA4* fall into the PR state and are enriched for H3K27me3 in fetal chondrocytes, while the active marks are absent. In undifferentiated H1-hESCs, the *SOX9* locus has a bivalent signature as previously reported^45^; this state resolves to active as cells are directed toward cartilage specification (Fig. 6c). Taken together, these findings further corroborate the accuracy and relevance of our gene modules and affirm that epigenetic regulation is intrinsically tied to human fetal skeletogenesis. Furthermore, they can serve as a resource to guide future studies focused on understanding the epigenetic regulation of individual genes important for musculoskeletal development.

### Comparison of in vivo and in vitro cartilage maturation

**Fig. 7:**
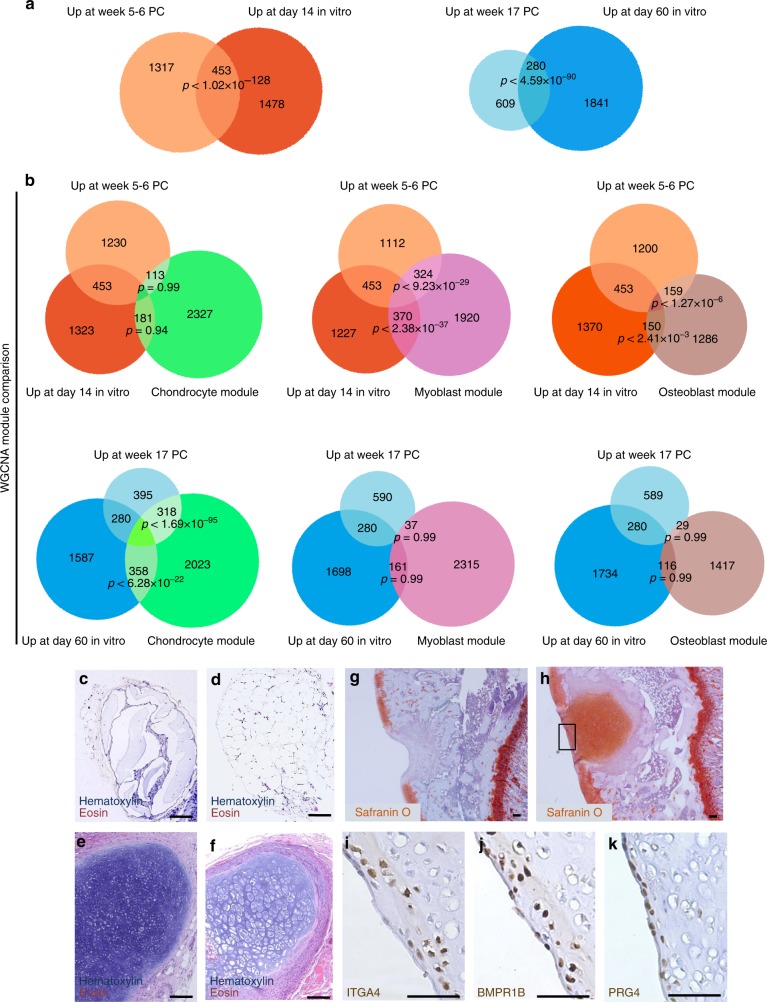
In vitro chondrogenesis strongly resembles in vivo chondrocyte development. **a** Overlaps of genes significantly differentially enriched in early developmental stages (d14 in vitro and 5–6 WPC in vivo) and in later developmental stages (d60 in vitro and 17 WPC in vivo). **b** Inclusion of module-specific genes (chondrocyte, green; myoblast, pink; osteoblast, brown) defines the lineage relationship of overlapping genes in each comparison. Degrees of overlap should be compared between columns. **c**–**f** Representative hematoxylin and eosin stains of sections from chondrogenic cells injected into the flanks of nude rats (c = 5–6 WPC; d = d14; e = 17 WPC; f = d60). **g**–**k** Focal defects were created in rat articular cartilage and either filled with vehicle (**g**) or d60 PSC-derived chondrocytes suspended in vehicle (**h**–**k**) and harvested after 30 days. Representative images of staining for the indicated makers is shown. *N* = 3; scale bars = 50 μm

To further characterize the degree of similarity between fetal cartilage development and our in vitro differentiation protocol, we compared the genes differentially expressed between embryonic and fetal cartilage to those differentially expressed between d14 and d60 of in vitro differentiation (Fig. 7a). This approach identified a very significant overlap between the genes enriched at earlier stages in both systems (week 5–6 and d14) as well as those genes enriched later (17 WPC and d60; Fig. 7a). Using GO analysis we identified significant enrichment for terms such as cell adhesion and migration, ECM organization and disassembly, glycosaminoglycan metabolic process, cartilage development and collagen catabolic process at d60/week 17 (Supplementary Data 34). These data suggest that cells at these stages are generating substantial matrix and undergoing active migration and tissue remodeling. Conversely, the GO terms enriched at d14/week 5–6 were indicative of broader developmental potential, including terms such as epithelium, nervous system and cardiovascular development (Supplementary Data 35). In contrast to stage-matched comparisons, there was minimal overlap when comparing early and late stages (Supplementary Figure 8a, b).

To gain further insight into the cartilage specification process both in vivo and in vitro, we applied the modules defined by WGCNA to these data. Comparison of the genes enriched early in chondrogenic cells (week 5–6 and d14) with the chondrocyte module (Fig. 2c) resulted in minimal, non-significant overlap. However, intersection of these genes with the osteoblast and myoblast modules resulted in strongly significant overlaps (Fig. 7b), suggesting that at this early stage of chondrogenic commitment, there remains a substantial level of transcription unrelated to chondrogenesis in both the embryonic limb bud and in PSC-derived cells. In contrast, comparison of genes enriched later in chondrogenesis (week 17 and d60) with the chondrocyte module presented a very significant overlap while there was little overlap with the osteoblast and myoblast modules (Fig. 7b). Taken together, these findings demonstrate the utility of the human fetal musculoskeletal gene modules as a tool for defining the extent and fidelity of an in vitro differentiation process. Moreover, our data suggest that the transcriptional promiscuity often maligned in PSC derivatives is largely representative of in vivo lineage specification during human development.

On the basis of our transcriptional analyses, we hypothesized that transcriptional instability during specification may represent a continued need for exogenous signaling to maintain chondrocyte commitment and/or promote maturation. Accordingly, we transplanted primary human pre-chondrocytes isolated at 5–6 WPC of embryogenesis (Fig. 7c) as well as PSC-derived cartilage-committed cells at d14 into the flanks of nude rats (Fig. 7d). Neither primary nor PSC-derived cells from these stages were able to engraft and form cartilage anlagens. However, week 17 primary chondrocytes (Fig. 7e) or d60 PSC-derived cartilage (Fig. 7f) were both capable of forming cartilage anlagens, demonstrating that cellular maturation is necessary for functional chondrogenesis. Interestingly, cultured d60 PSC-derived chondrocytes are enriched for ITGA4^+^BMPR1B^−^ cells, with a minority of more differentiated cells expressing both BMPR1B and ITGA4 (Supplementary Figure 8c). This expression profile is typical for primary mesenchymal cells at the initial phase of cartilage condensation (Supplementary Figures 4a–f) while emerging chondrocytes also express BMPR1B^24^. Implantation of d60 chondrocyte aggregates into focal osteochondral defects in the rat distal femur resulted in robust engraftment and formation of COL2^+^ human cartilage (Supplementary Figures 8d, e) with appropriate expression of proteoglycans (Fig. 7g, h), although it cannot be excluded that some endogenous rat cells are present around the borders of the implant. We also observed expression of PRG4 specifically in the most superficial cells, with ITGA4^+^ and BMPR1B^+^ cells localized to the superficial zone (Fig. 7i–k, Supplementary Figures 8f–h), similar to the zonal architecture of normal adult articular cartilage. Importantly, the vast majority of implanted human cells became ITGA4 negative in vivo, similar to mature chondrocytes in the deeper layers of native articular cartilage. These data suggest that implanted PSC-derived chondrocytes receive cues from the local microenvironment for proper structural organization. Together, these findings show that in vitro chondrogenesis from PSCs closely mimics human development and represent an in silico “molecular scale” to grade the potential functional output from future differentiation protocols for cartilage and other musculoskeletal tissues.

## Discussion

Here, we generated data sets to interrogate lineage specification and maturation during human musculoskeletal development, as well as during in vitro differentiation of human PSCs into chondrocytes. Our results demonstrate a surprising amount of transcriptional “noise” following cartilage specification in vivo, with genes of multiple musculoskeletal lineages undergoing progressive silencing with development. Generation of chondrocytes from PSCs was markedly similar, with cells isolated at earlier stages expressing many of the same genes from other lineages found during pre-chondrocyte specification at 5–6 WPC; additional differentiation in vitro not only mirrored the silencing of these genes observed in vivo, but also resulted in the generation of cells with similar functionality to human fetal presumptive articular chondrocytes at 17 WPC. Moreover, interrogation of these data sets enabled the definition of functionally heterogeneous populations in adult pig articular cartilage, including a sub-population possessing unique osteochondral potential. These results demonstrate the utility of this resource in understanding the normal biological processes that occur as cells undergo progressive specification and lineage commitment during human development.

Longitudinal analysis of human chondrogenesis across 4 unique stages provides a detailed picture of how chondrocytes are specified and mature. We identified a transcriptional program that is shared early in embryonic and fetal development and furthermore noted significant similarities between adolescent and adult cartilage, while also observing differences which distinguish the two post-natal stages. Two recent studies investigating human juvenile articular cartilage by microarray analysis identified a number of interesting genes that were enriched in the chondrocyte module (Supplementary Data 3), including *UCMA, ETV5, MATN1* and *MSMP*. *TFC2PL1*, a transcription factor whose motif and expression are enriched in the chondrocyte module (Supplementary Data 3 and 8), was also identified. This gene was recently shown to be a critical target of β-catenin^46^ that regulates self-renewal and pluripotency in human PSCs, but its role in chondrocytes remains unclear. *CHRDL1* was also identified as a potential regulator of proliferation in juvenile samples^47^. Although *CHRDL1* was not enriched in our adolescent vs. adult comparison, our data also indicate an increased capacity for proliferation in adolescent chondrocytes compared to adult cells. Taken together, these data lend increased molecular support for the advancement of therapies utilizing juvenile chondrocytes (particulated juvenile articular cartilage, PJAC) in allografts to treat focal defects in cartilage^48,49^.

Analysis of our longitudinal cartilage specification and maturation data identified ITGA4 as a marker of a rare chondrocytes possessing more primitive characteristics than other cells present in adult articular cartilage. Integrins are known to be highly expressed in cartilage as they facilitate cell-ECM interactions. ITGA5, which along with ITGA4 binds to fibronectin^50^ following heterodimerization, has the highest abundance in cartilage (Supplementary Figure 3b); in contrast, ITGA10, which has previously been identified as a potential marker for chondrocyte progenitors in bovine cartilage^35^, has specificity for COL2^51^. Interestingly, ITGA4 expression has been previously studied in human cartilage pathology where it was shown to be upregulated specifically in osteoarthritis (OA), while it was reported to be absent in healthy cartilage^52^. These data may suggest that ITGA4^+^ chondrocytes either proliferate in diseased joints or that ITGA4^−^ cells turn on *ITGA4* under certain conditions. Further studies will be required to elucidate the function of ITGA4 in development and disease, as well as the dynamics of chondrocyte populations in articular cartilage.

We and others have previously reported generating articular-like chondrocytes from PSCs^24,53–55^. Although Lee et al. assessed gene expression of PSC-derived chondrocytes by microarray at multiple stages of differentiation, these data were only compared to adult articular chondrocytes; cells cultured the longest were found to be the most similar to adult cells^54^. The concept that extended culture improves maturation of PSC-derived chondrocytes was also demonstrated by Craft et al.^55^. In the current work, we demonstrate transcriptional plasticity during in vivo chondrocyte specification and identify similarities with PSC-derived chondrocytes at two stages of differentiation by RNA and ChIP-sequencing. This molecular roadmap for chondrocyte ontogeny could accelerate tissue engineering and disease-modeling applications. Moreover, our data define a strong overlap in gene expression between ligamentocytes derived from intra-synovial cruciate ligaments and chondrocytes at 17 WPC suggesting chondrogenic differentiation protocols for PSCs could also be adapted to produce ligamentocytes for potential therapeutic applications.

The data presented here will serve as a foundation to understand critical molecular steps in human development. We have already applied these findings to directed differentiation of PSCs to cartilage for tissue engineering, and our collaborators have identified ERBB3 and NGFR from the muscle module as novel markers of muscle progenitors during human development and in vitro PSC differentiation^32^. These results support the necessity and value of this resource, which will continue to provide molecular insight for tissue engineering, disease-modeling and regenerative medicine applications.

## Methods

### General methods

For all experiments, biological replicates were employed to generate data. For experiments expected to yield large differences, standard practice of using 3 replicates was followed. All statistical methods are described in the figure legends.

### Tissue collection and digestion

Adult human primary tissue samples were obtained from National Disease Research Interchange (NDRI). Fetal and embryonic tissue samples were obtained from Novogenix Laboratories. All donated material was anonymous, carried no personal identifiers and was obtained after informed consent. Sex of the specimens was unknown. Pig cartilage and synovium were harvested from pig legs supplied by S&S Farms. Human and pig primary tissues were manually cut into small pieces and digested 4–16 h at 37 °C with mild agitation in digestion media consisting of DMEM (Corning) with 10% FBS (Sigma), 1 mg/mL dispase (Gibco), 1 mg/mL type 2 collagenase (Worthington), 10 –µg/mL gentamycin (Teknova) and primocin (Invivogen). Mouse cartilage was digested for 3–4 h and then sorted.

### Antibody list

Please see Table 1 for a list of antibodies and dilutions used.

**Table 1:** Antibodies used in this study

Antibody	Vendor	Catalog number	Dilution
CD45-FITC	BD Biosciences	555482	20 μL/10^6^ cells
CD31-FITC	BD Biosciences	555445	20 μL/10^6^ cells
CD235a-FITC	BD Biosciences	559943	0.5 μL/10^6^ cells
CD146-PE	BD Biosciences	550315	20 μL/10^6^ cells
ALP-PerCP-Cy5.5	BD Biosciences	561508	5 μL/10^6^ cells
CD144-PE	BD Biosciences	560410	20 μL/10^6^ cells
CD56-APC	Biolegend	318310	10 μL/10^6^ cells
CD73-APC	BD Biosciences	560847	5 μL/10^6^ cells
CD34-PerCP-Cy5.5	BD Biosciences	347222	20 μL/10^6^ cells
CD166-PE	BD Biosciences	560903	20 μL/10^6^ cells
CD146-PECy7	BD Biosciences	562135	5 μL/10^6^ cells
CD44-PerCP-Cy5.5	BD Biosciences	560531	5 μL/10^6^ cells
CD309-PE	R&D Systems	FAB357P	10 μL/10^6^ cells
CD326-PE	BD Biosciences	347198	20 μL/10^6^ cells
CD166-PE	BD Biosciences	559263	20 μL/10^6^ cells
ITGA4-PECy7	Biolegend	304314	2 μL/10^6^ cells
Ter119-PE	Biolegend	116208	1 μL/10^6^ cells
CD45-PE	Biolegend	103106	1 μL/10^6^ cells
CD31-PE	Biolegend	102508	5 μL/10^6^ cells
BMPR1B-APC	R&D Systems	FB5051A	2 μL/10^6^ cells
CD56	Abcam	ab9018	1:100 (IHC)
Collagen II	Abcam	ab185430	1:250 (IHC)
GFP	Abcam	ab13970	1:500 (IHC)
CD146	Abcam	ab215884	1:100 (IHC)
BMPR1B	Thermo	PA5-11861	1:250 (IHC)
PRG4	Abcam	ab28484	1:250 (IHC)
ITGA4	Bioss	bs-0641R	1:250 (IHC)
GLI1	Bioss	bs-1206R	1:1000 (WB)
pSTAT3 (Tyr705)	Cell Signaling	9145	1:1000 (WB)
SOX9	Abcam	ab26414	1:1000 (WB)
RUNX2	Abcam	ab48811	1:1000 (WB)
Histone H3	Cell Signaling	4499	1:1000 (WB)
H3K4me3	Diagenode	C15410003-50	1 μg/IP
H3K4me1	Diagenode	C15410194	1 μg/IP
H3K27ac	Diagenode	C15410196	1 μg/IP
H3K27me3	Diagenode	C15410195	1 μg/IP
Anti-Mouse IgG ImmPRESS	Vector	MP-7422	Pre-diluted
Anti-Rabbit IgG ImmPRESS	Vector	MP-7401	Pre-diluted
Anti-Chicken HRP	Abcam	ab6897	1:500
Anti-Rabbit DyLight 488	Biolegend	406404	1:500
Anti-Mouse DyLight 594	Abcam	ab96873	1:500

### Flow cytometry and FACS

For FACS, cells were blocked in 1% FBS in PBS or human IgG (Invitrogen; 1 µg for 10^6^ cells) for 10 min before a 20 min incubation with primary antibodies. Cells were then washed twice in 1% FBS and stained with DAPI for viability. Populations of interest were directly sorted into DMEM with 10% FBS. 17 wk fetal cells were sorted according to Fig. 1. Adolescent and adult chondrocytes were sorted following digestion as BMPR1B^+^CD34^−^LIN^−^ cells. PSC-derived chondrocytes for RNA-Seq were sorted as BMPR1B^+^CD166^−^. Mouse articular chondrocytes were sorted from 2 month old male B6 animals as CD31^−^CD45^−^Ter119^−^ALP^−^BMPR1B^+^.

### Immunohistochemistry (IHC)

Tissues were fixed in 10% formalin and sectioned at 5 μm^24^. For DAB, sections were deparaffinized using standard procedures and antigen retrieval was performed by bringing samples to a boil in 1x citrate buffer pH 6.0 (Diagnostic Biosystems), and incubating at 60 degrees Celsius for 30 min followed by 15 min cooling at room temperature. Endogenous peroxidase activity was quenched by treating samples with 3% H_2_O_2_ for 10 min at RT. Sections were then blocked in 2% normal horse serum for 20 min. Sections were then incubated with primary antibodies diluted in TBS with 1% BSA (Sigma) overnight at 4 degrees Celsius. Sections were washed 3 times with TBS .05% Tween 20 (Sigma) (TBST) before addition of HRP-conjugated secondary antibody for 30 min incubation at RT. Sections were washed 3 times with TBST after secondary incubation and DAB substrate was then added until positive signal was observed. Sections were then immediately washed with tap water, counterstained in hematoxylin for 30 s and washed again with tap water before dehydration and mounting. For Hematoxylin and eosin staining, sections were deparaffinized, rinsed in tap water, and stained with Hematoxylin for 3 min. Sections were then washed in tap water, and stained with Eosin for 2 min before a final wash in tap water.

For immunofluorescence of human sections, slides were deparaffinized, and antigen retrieval was performed as described above. Sections were then rinsed twice with TBST and blocked in normal horse serum for 30 min at RT. Sections were incubated in primary antibody in TBS 1% BSA overnight at 4°C. Sections were washed twice in TBST before addition of fluorophore conjugated secondary antibody in TBS 1% BSA for 30 min at RT in the dark. Sections were washed twice in TBST and then counterstained with DAPI for 15 min at RT. Sections were washed once before mounting with anti-fade medium and imaging. Immunofluorescence on mouse and pig sections was performed with tyramide kits (Thermo) following manufacturer’s instructions.

### Differentiation of human pluripotent stem cells to cartilage

Differentiation of cartilage from human PSCs was conducted as follows. H1-hESCs maintained by UCLA were cultured in mTESR media before addition of Mesoderm Induction (MI) media 1 (MI-1): X-Vivo (Lonza), FGF2 (10 ng/ml), Activin A (10 ng/ml), Wnt3a (10 ng/ml). After 3 days this medium was refreshed and Noggin (50 ng/ml) was added to MI-1 to minimize specification of cardiovascular lineage and Activin A removed to minimize endoderm formation. Cells were cultured in MI-1 with Noggin for additional for 4 days. Next, Chondrogenic Induction media A (CI-A) was added: BMP4 (10 ng/ml), FGF2 (10 ng/ml) for 3 days. On Day 10–11 epithelial endodermal, cardiovascular and hematoendothelial mesodermal cells as well as undifferentiated PSCs were depleted by Magnetic Assisted Cell Sorting (MACS) using CD326/EpCAM and CD309/VEGFR2 antibodies (Miltenyi Biotech)^24^. After MACS depletion, cells were recovered in regular culture flasks for 3 days in Chondrogenic Induction media A (CI-A) supplemented with Rock inhibitor Y27632 (Tocris, 10 nM), followed by Chondrogenic Induction media B (CI-B): FGF2 (10 ng/ml), SHH (50 ng/ml), BMP4 (50 ng/ml) for 3 days and transferred to a hypoxic chamber (Biospherix) and kept at 5% O_2_. At this point, ~200,000 cells were seeded in each well of an ultra^−^low attachment 96 well plate (Nunc) to form chondrogenic aggregates; After 3 days in CI-B, media was removed and transferred to Maintenance Media (MM): X-Vivo, IGF2 (10 ng/mL), FGF2 (10 ng/mL), LIF (50 ng/mL), TGF-β1 (10 ng/mL), BMP4 (1 ng/mL). At this timepoint (Day 14) some aggregates were harvested for RNA-seq analysis. Remaining aggregates were cultured for up to 60 days and then analyzed using histological techniques, FACS, Chip-Seq and RNA-Seq. Media was changed every 7 days until day 60. All growth factors were obtained from Peprotech and R&D Systems. H1 cells were routinely tested for mycoplasma during maintenance.

### RNA-seq library preparation and sequencing

RNA was isolated using QIAGEN RNeasy Micro Kits. Prior to library preparation, RNA was quantified using Qubit fluorometer (Thermo Fisher Scientific), and run on Agilent Bioanalyzer 2100 for quality control. For primary human and mouse tissue samples, the Nugen Ovation RNA-Seq System V2 kit was used to generate double-stranded cDNA using a mixture of random and poly(T) priming. Kapa LTP library kit was used to make the sequencing library. The workflow consists of fragmentation of double-stranded cDNA, end repair to generate blunt ends, A-tailing, adaptor ligation and PCR amplification. Different adaptors were used for multiplexing samples in one lane. Sequencing was performed on Illumina HiSeq 2500 with single-end 50 base pair reads. Data quality check was done on Illumina SAV. Demultiplexing was performed with Illumina CASAVA 1.8.2.

For pig chondrocytes, library preparation was performed using the NuGen Ovation SoLo RNA-Seq system according to manufacturer’s protocol. Briefly, Total RNA was treated with HL-dsDNase and first strand cDNA synthesis was performed. cDNA was then processed before second strand synthesis. End repair was conducted before Adaptor ligation. Libraries were then amplified based on starting RNA input. Strand selection and adaptor cleavage was performed before a second Library Amplification. Quality of libraries was assessed using Agilent Bioanalyzer 2100. Sequencing was performed on Illumina HiSeq 2500 with a paired-end 100 bp run. Data quality check was done on Illumina SAV. Demultiplexing was performed with Illumina CASAVA 1.8.2.

### WGCNA

Network construction and module detection were performed using default settings as described^28^.

### Differential expression analysis

For human and mouse samples, transcript levels were estimated using RNA-Seq by Expectation Maximization (RSEM^56^). Reads were mapped to the human (hg19 or hg38) or mouse GRCm38 (mm10) genomes using RefSeq annotations. Transcript per million (TPM) expression estimates were transformed to log2(TPM + 1) to weigh against inflated counts. Pairwise differential expression assessments were performed using EBSeq^57^. For pig samples, reads were aligned to the Sus-scrofa_Ensembl84 reference using STAR aligner. Transcript levels were quantified to the reference using Partek E/M using standard input parameters; genes were considered to be differentially expressed based on a False Discovery Rate of less than 5% (Posterior Probability of being Differentially Expressed < 0.05). Gene Ontology (GO) analysis was performed using DAVID; all p values listed are a modified Fisher’s Exact test (EASE). Heat maps were generated by calculating the log ratio expression value of each group versus the average expression value of all samples for each gene^58^. Two and 3-way Venn diagrams were generated using BioVenn^59^, while 4- and 5-way Venn diagrams were constructed using http://bioinformatics.psb.ugent.be/webtools/Venn. Hypergeometric *p* values were calculated assuming 25,000 human genes.

### Transcription factor motif enrichment analysis

OPOSSUM 3.0^31^ was used to define motifs of known transcription factors enriched in sets of genes. Default setting were used for the analysis, with the exception of promoters being defined as 500 bp preceding and 100 bp following the TSS. Only motifs with a *Z* score > 10 and a Fisher score > 7 were considered enriched.

### Western blots

Sorted cells were lysed in RIPA Lysis and Extraction Buffer (Pierce) containing protease inhibitors (Pierce). Cell lysates were then sonicated with a 15-second pulse at a power output of 2 using the VirSonic 100 (SP Industries Company). Protein concentrations were next determined with a BCA protein assay (Pierce). Proteins were resolved with SDS-PAGE utilizing 4–15% Mini-PROTEAN TGX Precast Gels (Biorad) and transferred to Trans-Blot Turbo Transfer Packs (Biorad) with a 0.2-µm pore-size nitrocellulose membrane (Biorad). Nitrocellulose membranes were blocked in 5% nonfat milk in 0.05% (v/v) Tween 20 (PBST) (Corning). Membranes were then incubated with primary antibodies overnight. After washing in PBS containing 0.05% (v/v) Tween 20 (PBST), membranes were incubated with secondary antibodies (Thermo Scientific). After washing, membranes were developed with the Clarity Western ECL Blotting Substrate (Biorad). Histone H3 was used as a loading control for all blots. Please see Supplementary Figure 9 for uncropped scans of representative blots.

### Chromatin IP

ChIP was performed with 10,000 FACS-sorted cells per IP using Diagenode Low Cell ChIP kit (cat# C01010072) according to manufacturer’s protocol. 1 µg of corresponding antibody from Diagenode was used per IP: H3K4me3 (cat# C15410003-50, lot# A5051-001P), H3K4me1 (cat# C15410194, lot# A1862D), H3K27ac (cat# C15410196, lot# A1723-0041D), H3K27me3 (cat# C15410195, lot# A1811-001P).

### ChIP library preparation and sequencing

ChIP-seq libraries were constructed using Nugen’s Ovation Ultralow DR Multiplex System 1–8 (cat# 0330-32) according to manufacturer’s instructions. Library quality and concentration was assessed using Agilent Bioanalyzer 2100 and Qubit (Thermo). Libraries were sequenced on Illumina HiSeq2000 platform. After initial quality control the demultiplexed reads were aligned to the human genome GRCh37 (hg19) using bowtie v0.12.7^60^ with (-m 1 –strata –best –v 2) parameters.

### ChromHMM analysis

A joint ChromHMM model^44^ was learned by using ChIP-seq data for H3K4me1, H3K4me3, H3K27ac, H3K27me3 and Input for H1-hESC, H1-14d, H1-60d, Fetal and Adult. Data for H1-hESC was downloaded from the Roadmap Epigenomics project^61^ from: http://egg2.wustl.edu/roadmap/data/byFileType/alignments/consolidated.

For the rest of the cell types, the aligned sequencing reads were pooled for all biological replicates for each mark produced within the study. ChromHMM default options were used to binarize the pooled reads while treating the Input reads as control. ChromHMM models were explored with varying numbers of chromatin states in the range between 2 and 16. A 12 state model was picked for our analysis because it was the model with the minimum number of states that captured a poised enhancer state, whereas models with more states did not seem to capture additional biologically relevant states. All enrichments in Fig. 6a (right panel) and 6B were calculated on base level as the ratio between the observed and the expected overlap between the corresponding pair of genomic features. The expected overlap was computed based on a random binomial model where the two features are treated as independent. The enrichments in the right panel of Fig. 6a are based on the genome-wide background distribution of chromatin states. The enrichments in Fig. 6b are based on the background distribution of chromatin states in promoter proximal regions defined as TSS ± 2 kb intervals at all annotated RefSeq TSSs.

Genes enriched for particular chromatin states (AP, PP and PR) in Supplementary Figures 6b–d are defined as genes whose promoter proximal regions (TSS ± 2 kb) have a significantly higher number of genomic bins annotated with the chromatin state compared to promoter proximal regions of all genes in the fetal sample. The enrichment is based on a binomial test at false discovery rate of 0.05.

### Quantitative real-time PCR

Power SYBR Green (Applied Biosystems) RT-PCR amplification and detection was performed using an Applied Biosystems Step One Plus Real-Time PCR machine. The comparative Ct method for relative quantification (2-ΔΔCt) was used to quantitate gene expression. TBP (TATA-box binding protein) was used for gene normalization and expressed relative to a calibrator (sample in each set with lowest expression). Primer sequences used for qPCR are available on request.

### Osteogenic and chondrogenic differentiation assays

FACS-sorted populations were plated in DMEM with 10% FBS and expanded for one passage before re-seeding and addition of appropriate differentiation media (STEM-PRO, Invitrogen). For osteogenesis, 10,000 cells were plated in each well of a 12 well plate (Corning). For the chondrogenic pellet assay, 100,000 cells were added to a 15 mL Falcon tube (Corning) and spun down at 500 RPM for 5 min at 4 °C in 2 mL media. Cells were treated in differentiation media for two weeks with media changed after one week of treatment.

### Alizarin red stain

Cells were fixed with 1 mL 10% Formalin for 10 min at RT. Fixed cells were then washed twice with PBS before staining with 1 mL of 2% Alizarin Red S solution (ScienCell cat#8678a) for 30 min at RT. Dye was removed and washed 5X with diH_2_0. Alizarin red staining was quantified by addition of 400 μL 10% acetic acid to each well. Cells and solution were collected and vortexed in 1.5 mL microcentrifuge tube for 30 s. Samples were then heated at 85° Celsisus for 10 min before incubation on ice for 5 min. Samples were next centrifuged at 20,000 *g* for 15 min. A concentration of 200 μL of supernatant was transferred to a new tube and 75 μL of 10% ammonium hydroxide was added to neutralize acid. Absorbance was read at 405 nm with a plate reader.

### Alcian blue stain

Chondrocyte pellets were fixed for 15 min in 1 mL 10% Formalin and washed twice with PBS. Pellets were embedded in paraffin and sectioned at 5 µm for IHC. Sections were stained with Alcian blue for 30 min at room temperature and washed twice with diH_2_O before imaging. Quantification was performed with the Fiji ImageJ package^62^.

### Implantation of human chondrocyte populations

All experiments involving rats were conducted under the supervision of UCLA DLAM and USC DAR. Prior to implantation, primary pre-chondrocytes from enzymatically digested 5–6 week old human limbs were isolated using FACS and re-aggregated in ultralow attachment plates (Nunc)^63^. Fetal cartilage was dissected from 17-week old fetal tissues, enzymatically digested and re-aggregated for up to 48 in vitro. PSC-derived chondrogenic aggregates were generated as described above from a stable line generated in the Evseenko lab expressing GFP^64^. Primary and PSC-derived cell aggregates were then implanted sub-cutaneously into 6, 2 month old, athymic male rats (Harlan labs); no randomization nor blinding was used. 5 aggregates (~200,000 cells each) were implanted to each rat. Day 60 PSC-derived aggregates (2 per rat) were also implanted into full thickness - 1 mm in diameter—osteochondral defects generated in the knee joint of 6 athymic rats using the surgical procedure previously described by our group^65^. The chondrosphere aggregates were additionally fixed in place using EVICEL® Fibrin Sealant (Ethicon). After 4 weeks tissues were harvested and analyzed using histological techniques.

## Electronic supplementary material

Supplementary Information

Description of Additional Supplementary Files

Supplementary Data 1

Supplementary Data 2

Supplementary Data 3

Supplementary Data 4

Supplementary Data 5

Supplementary Data 6

Supplementary Data 7

Supplementary Data 8

Supplementary Data 9

Supplementary Data 10

Supplementary Data 11

Supplementary Data 12

Supplementary Data 13

Supplementary Data 14

Supplementary Data 15

Supplementary Data 16

Supplementary Data 17

Supplementary Data 18

Supplementary Data 19

Supplementary Data 20

Supplementary Data 21

Supplementary Data 22

Supplementary Data 23

Supplementary Data 24

Supplementary Data 25

Supplementary Data 26

Supplementary Data 27

Supplementary Data 28

Supplementary Data 29

Supplementary Data 30

Supplementary Data 31

Supplementary Data 32

Supplementary Data 33

Supplementary Data 34

Supplementary Data 35

Supplementary Data 36

## Data Availability

All data are deposited in GEO under the accession numbers GSE106292, GSE107592, GSE11849 and GSE11850. TPM values for all 17 wk tissues used to perform WGCNA are included in GSE106292. Total numbers of reads and mappable reads for all other samples are included in Supplementary Data 36.
