# Supplementary material for: Mapping molecular landmarks of human skeletal ontogeny and pluripotent stem cell-derived articular chondrocytes
Source: Nat Commun. 2018 Sep 7;9:3634. doi: 10.1038/s41467-018-05573-y (PMC6128860; doi:10.1038/s41467-018-05573-y)
Supplement: Supplementary file 1 — Supplementary Information [file 41467_2018_5573_MOESM1_ESM.pdf]

Mapping molecular landmarks of human skeletal ontogeny and pluripotent stem cell-derived articular chondrocytes

Gabriel B. Ferguson[#], Ben Van Handel[#], Maxwell Bay, Petko Fiziev, Tonis Org, Siyoung Lee, Ruzanna Shkhyan, Nicholas W. Banks, Mila Scheinberg, Ling Wu, Biagio Saitta, Joseph Elphingstone, A. Noelle Larson, Scott M. Riester, April Pyle, Nick M. Bernthal, Hanna K.A. Mikkola, Jason Ernst, Andre J. van Wijnen, Michael Bonaguidi, Denis Evseenko*

Correspondence to: evseenko@usc.edu

Supplementary Figures:

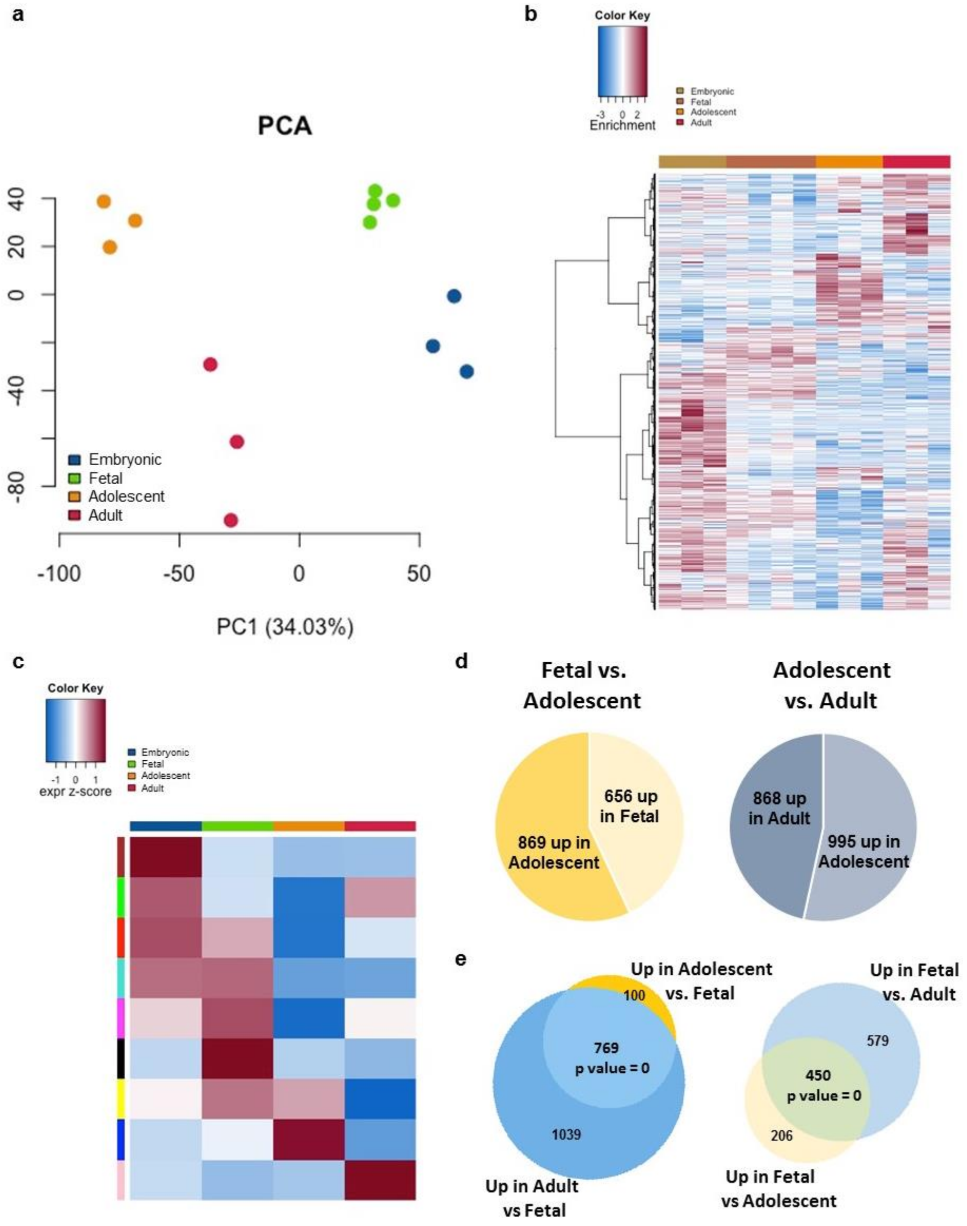

f

Up in Adolescent		Up in Adult	
GO Term	p value	GO Term	p value
Mitosis	3.35×10^{-7}	RNA processing	1.08×10^{-23}
Cell division	4.53×10^{-6}	RNA splicing	6.21×10^{-20}
Mitotic cell cycle	4.85×10^{-6}	Chromatin modification	4.18×10^{-7}
M phase	2.18×10^{-5}	Chromatin organization	6.48×10^{-5}
Extracellular matrix organization	1.49×10^{-4}	Regulation of gene expression, epigenetic	1.65×10^{-5}

Supplementary Figure 1. Adolescent cartilage closely resembles adult cartilage. (a) Principal Component Analysis (PCA) of 4 stages of human cartilage development. (b) Hierarchical clustering of 15,000 genes expressed across all 4 stages of human ontogeny. (c) WGCNA: Each column represents a different stage of human chondrogenesis and each row represents a unique gene module with relative gene expression levels portrayed by color scale. (d) Differentially expressed genes between fetal and adolescent stages and adolescent and adult stages. (e) Comparative analysis of differentially expressed genes between adolescent, adult and fetal stages identifies a highly significant overlap between those genes upregulated in both adolescent and adult stages compared to fetal. (f) GO analysis of genes differentially expressed between adolescent and adult cartilage. N = 3-4.

a

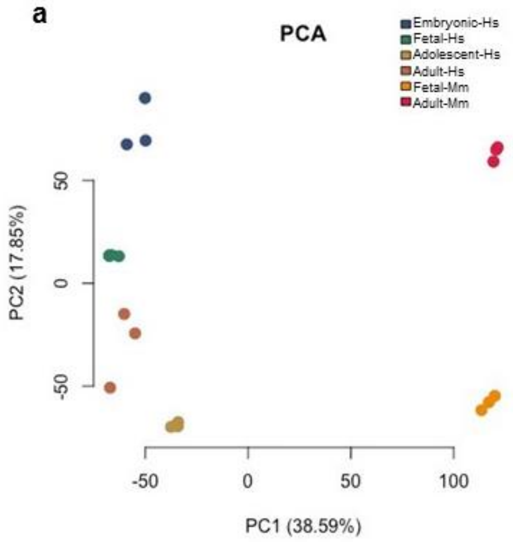

b

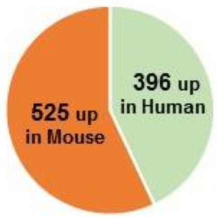

Up in Mouse		Up in Human	
GO Term	p value	GO Term	p value
Wnt receptor signaling pathway	$3.41x10^{-4}$	Electron transport chain	$6.25x10^{-8}$
Cell cycle	0.010	Cellular respiration	$3.86x10^{-7}$
MAPKKK cascade	0.014	Oxidative phosphorylation	$2.89x10^{-6}$
Insulin receptor signaling pathway	0.019	Cellular macromolecule localization	$1.12x10^{-4}$
Extracellular matrix organization	0.026	Ribosome biogenesis	$1.33x10^{-4}$

Supplementary Figure 2. Transcriptional comparison of human and mouse chondrocytes. (a) PCA plot demonstrating strong transcriptional differences driven by species. (b) Differentially expressed genes across all human and mouse samples with GO analysis. N = 3-4.

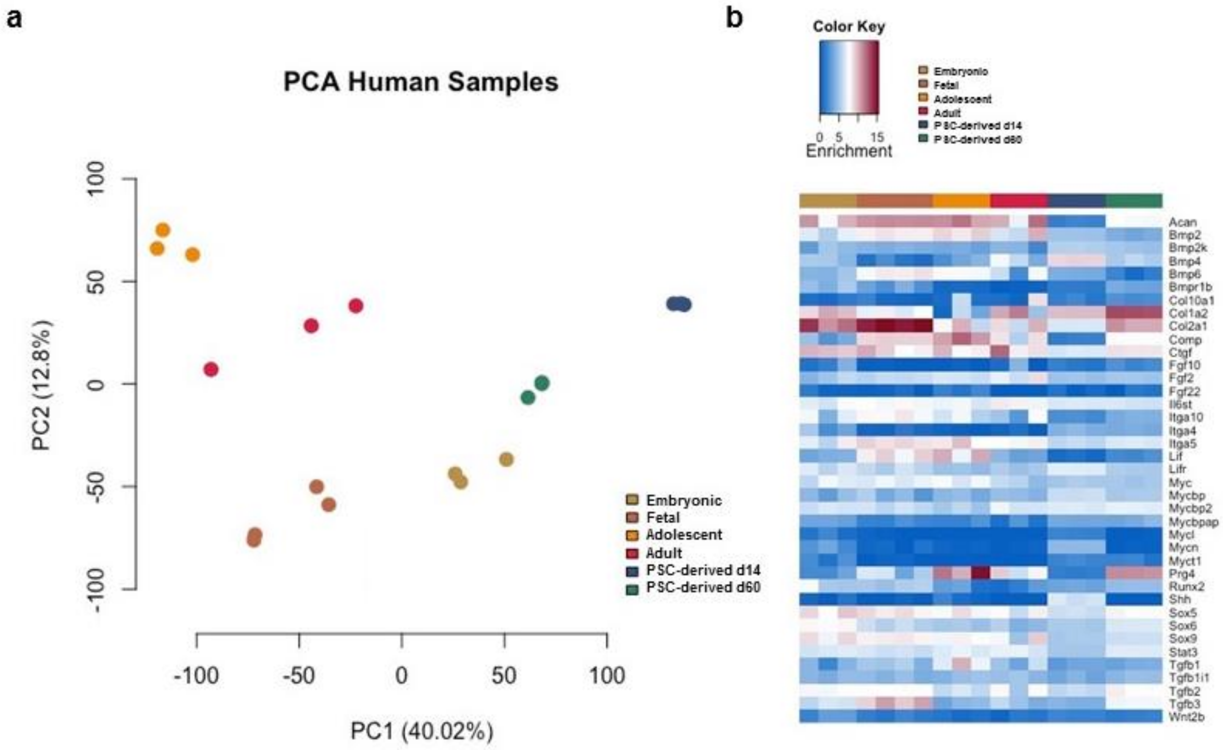

Supplementary Figure 3. Maturation of PSC-derived cartilage. (a) Principal Component Analysis (PCA) of 4 stages of human cartilage ontogeny and 2 stages of *in vitro* differentiated cartilage from human PSCs. (b) Heat map (log2 expression) of selected chondrogenic genes shown for all human *in vivo* and *in vitro* samples. N = 3-4.

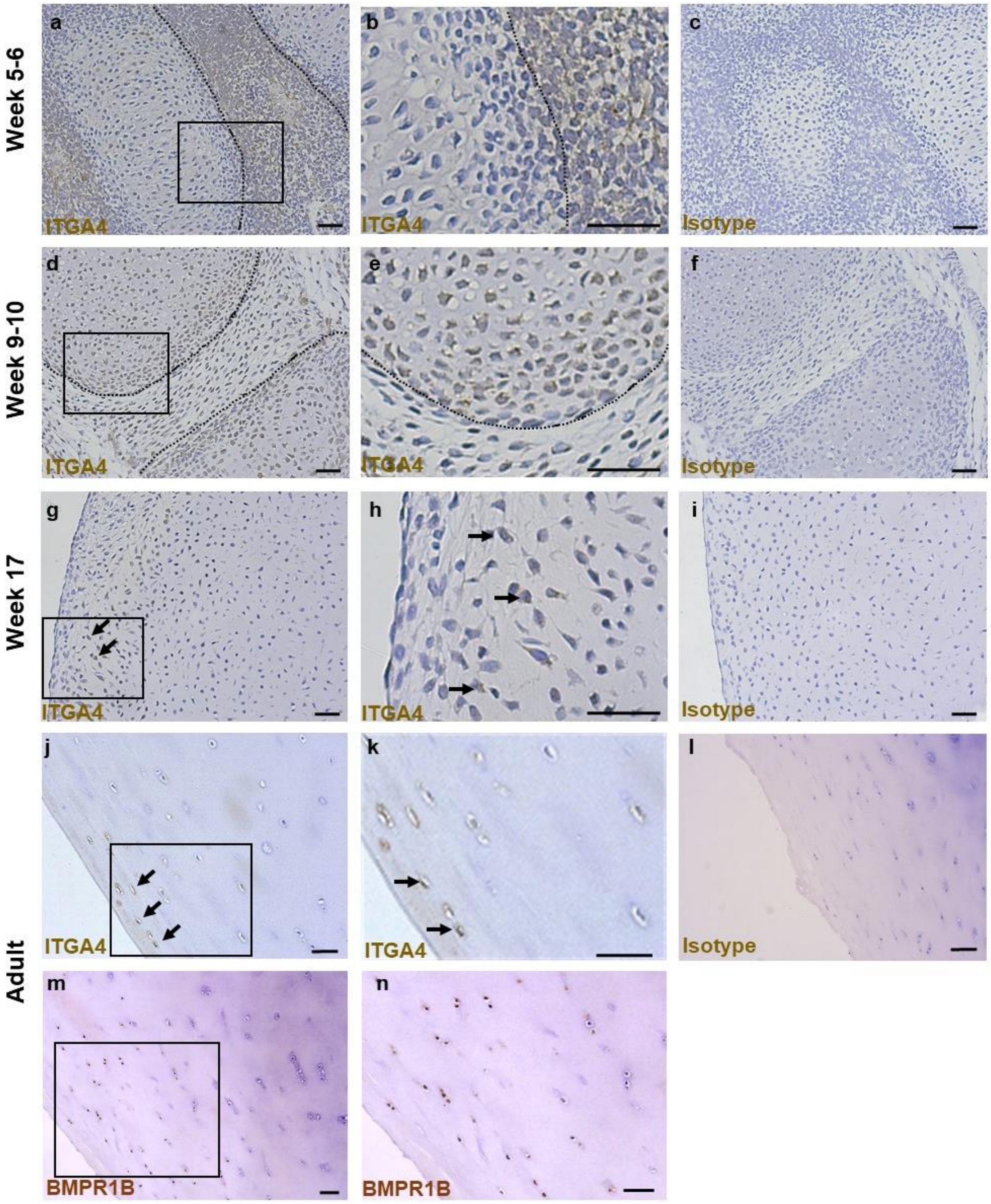

Supplementary Figure 4. Expression of ITGA4 in human chondrocytes. (a-c) Week 5-6 limb bud. (a-b) ITGA4 is strongly expressed and localized in mesenchymal condensations (dotted lines). (c) Isotype control. (d-f) Week 9-10. (d-e) ITGA4 is still widely expressed throughout mesenchyme. (f) Isotype control. (g-i) Week 17 fetal cartilage. (g-h) ITGA4 expression is barely detectable and restricted to a small percentage of superficial chondrocytes (arrows). (i) Isotoype control. (j-n) Adult articular cartilage. (j-l) ITGA4 expression is still restricted to a small number of superficial chondrocytes. (l) Isotype control. (m-n) BMPR1B is expressed into the transitional layer of articular chondrocytes, extending deeper than ITGA4. N = 3; scale bars = 50 um.

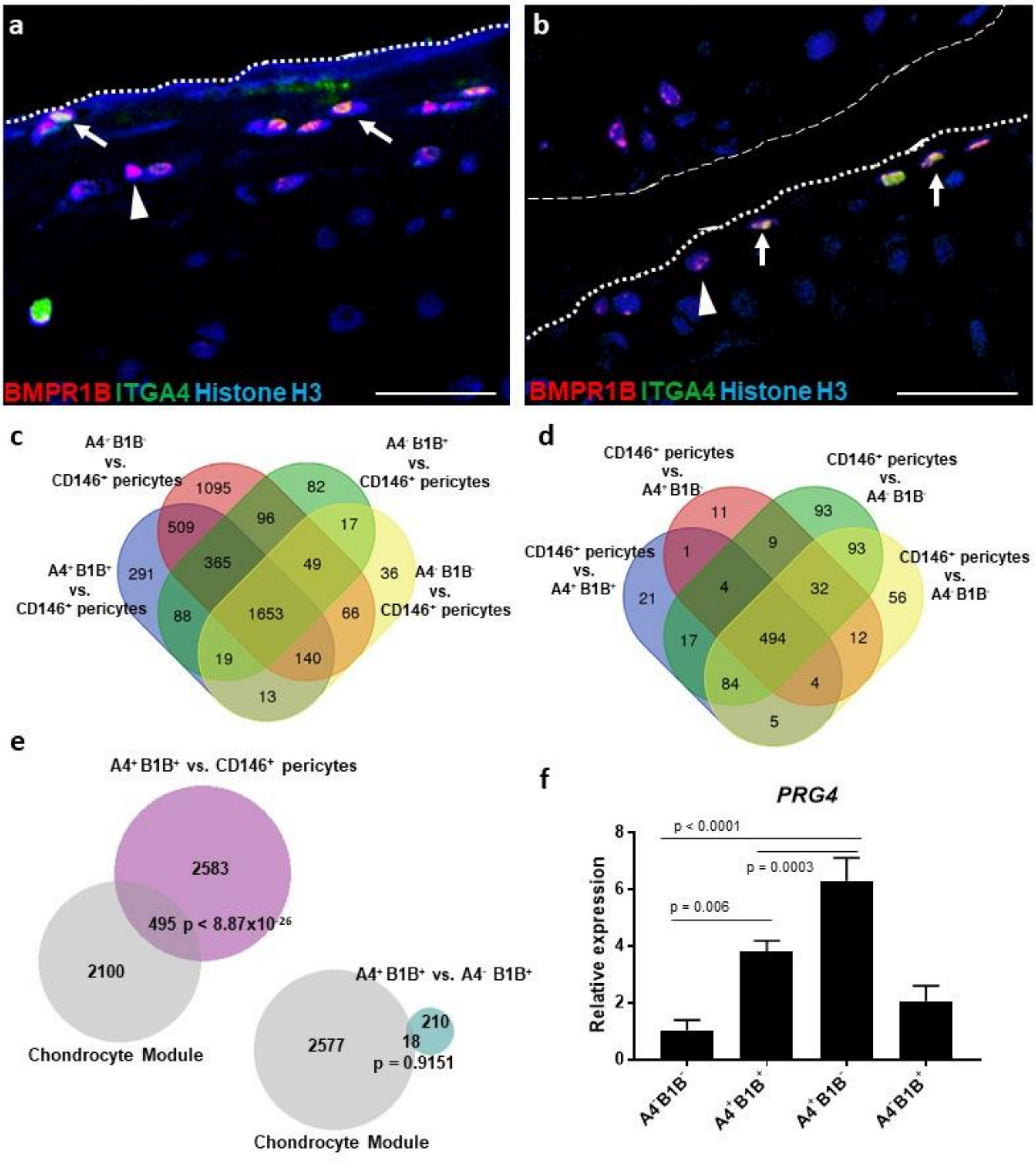

a
BMPR1B ITGA4 Histone H3
b
BMPR1B ITGA4 Histone H3
c
A4$^+$ B1B$^-$ vs. CD146$^+$ pericytes
A4$^-$ B1B$^+$ vs. CD146$^+$ pericytes
A4$^+$ B1B$^+$ vs. CD146$^+$ pericytes
A4$^-$ B1B$^-$ vs. CD146$^+$ pericytes
1095
82
509
96
17
291
365
49
36
88
1653
66
19
140
13
d
CD146$^+$ pericytes vs. A4$^+$ B1B$^-$
CD146$^+$ pericytes vs. A4$^-$ B1B$^-$
CD146$^+$ pericytes vs. A4$^+$ B1B$^+$
CD146$^+$ pericytes vs. A4$^-$ B1B$^-$
11
93
1
9
93
21
4
32
56
17
494
12
84
4
5
e
A4$^+$ B1B$^+$ vs. CD146$^+$ pericytes
2583
495 $p < 8.87 \times 10^{-26}$
2100
Chondrocyte Module
A4$^+$ B1B$^+$ vs. A4$^-$ B1B$^+$
2577
18
210
p = 0.9151
Chondrocyte Module
f
PRG4
Relative expression
p < 0.0001
p = 0.0003
p = 0.006
A4$^-$B1B$^-$
A4$^+$B1B$^+$
A4$^+$B1B$^-$
A4$^-$B1B$^+$

Supplementary Figure 5. ITGA4 and BMPR1B delineate articular cartilage subpopulations in pig. (a-b) Confocal images of the superficial layer of articular cartilage demonstrating expression of BMPR1B (red) and ITGA4 (green) in pig (a) and mouse (b). The dotted line indicates articular surface, and the dashed line demarcates meniscal tissue. Arrows indicate BMPR1B^{+}ITGA4^{+} cells; arrowheads identify BMPR1B^{+}ITGA4^{-} cells. (c-d) All-against-all comparison of genes significantly enriched in each pig articular chondrocyte population vs. CD146^{+} synovial pericytes (c) or in pericytes vs. the four populations (d). (e) Overlap of genes enriched in the cartilage module with genes differentially expressed between BMPR1B^{+}ITGA4^{+} pig chondrocytes and CD146^{+} pig synovial pericytes. For comparison, the overlap between the cartilage module and the 238 genes enriched in BMPR1B^{+}ITGA4^{+} vs. BMPR1B^{+}ITGA4^{-} chondrocytes is shown. (f) Quantitative PCR for *PRG4* expression across four pig chondrocyte populations. Data are represented as mean ± SD. Pooled data in panel f resulted from 3 biological replicates; significance was calculated using one-way ANOVA followed by Holm-Sidak's multiple comparison test. Error bars represent standard deviation. B1B = BMPR1B; A4 = ITGA4. N = 3; scale bars = 50 um.

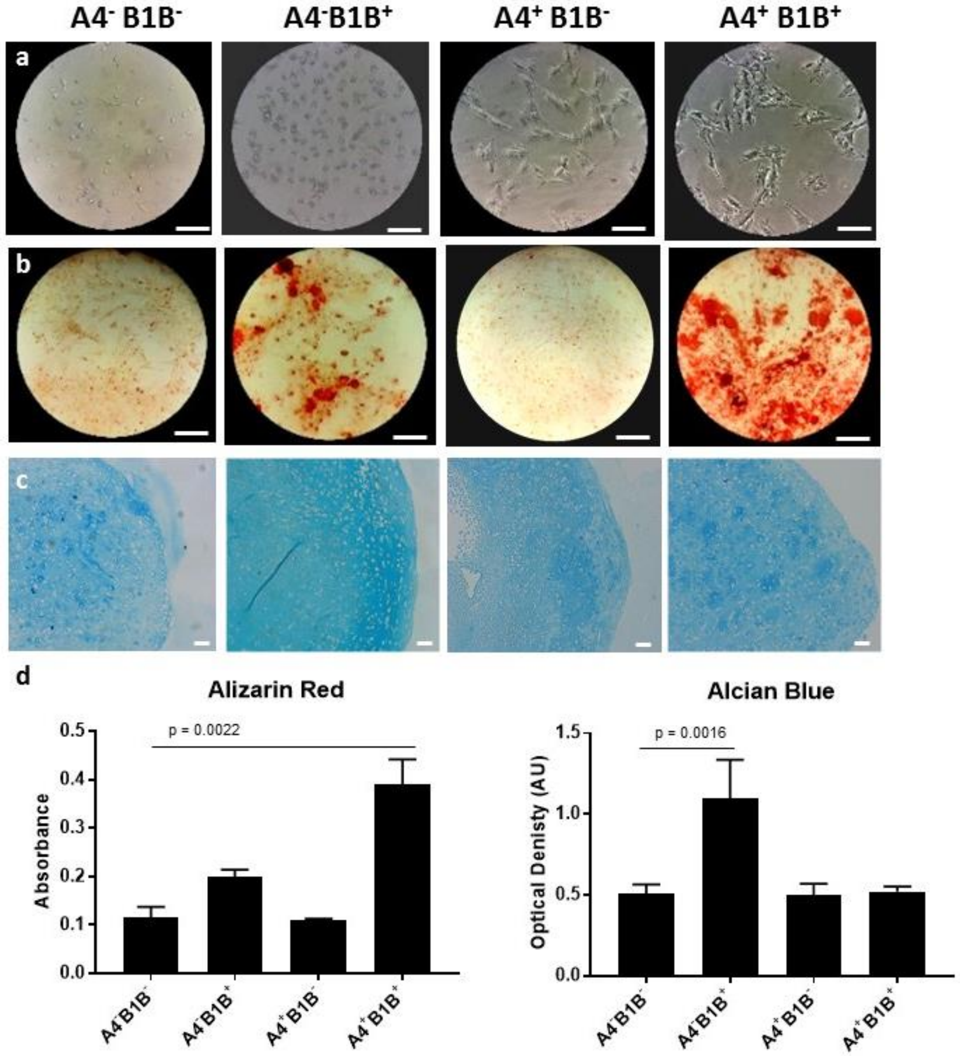

Supplementary Figure 6. Osteochondral potential of articular chondrocytes is defined by ITGA4 and BMPR1B. (a) Brightfield images of pig chondrocytes after one passage. Note the different morphologies of ITGA4^{+} vs. ITGA4^{-} cells. (b) Alizarin Red staining of cells treated with osteogenic media for 2 weeks. (c) Alcian Blue staining of sections from chondrogenic pellets cultured in chondrogenic media for two weeks. All images in A-D are representative of 3 independent replicates. (d) Quantification of osteo- and chondrogenic potential for each population. Error bars represent standard deviation. Pooled data in panel e resulted from 3 biological replicates; significance was calculated using one-way ANOVA followed by Holm-Sidak's multiple comparison test. N = 3; scale bars = 25 um.

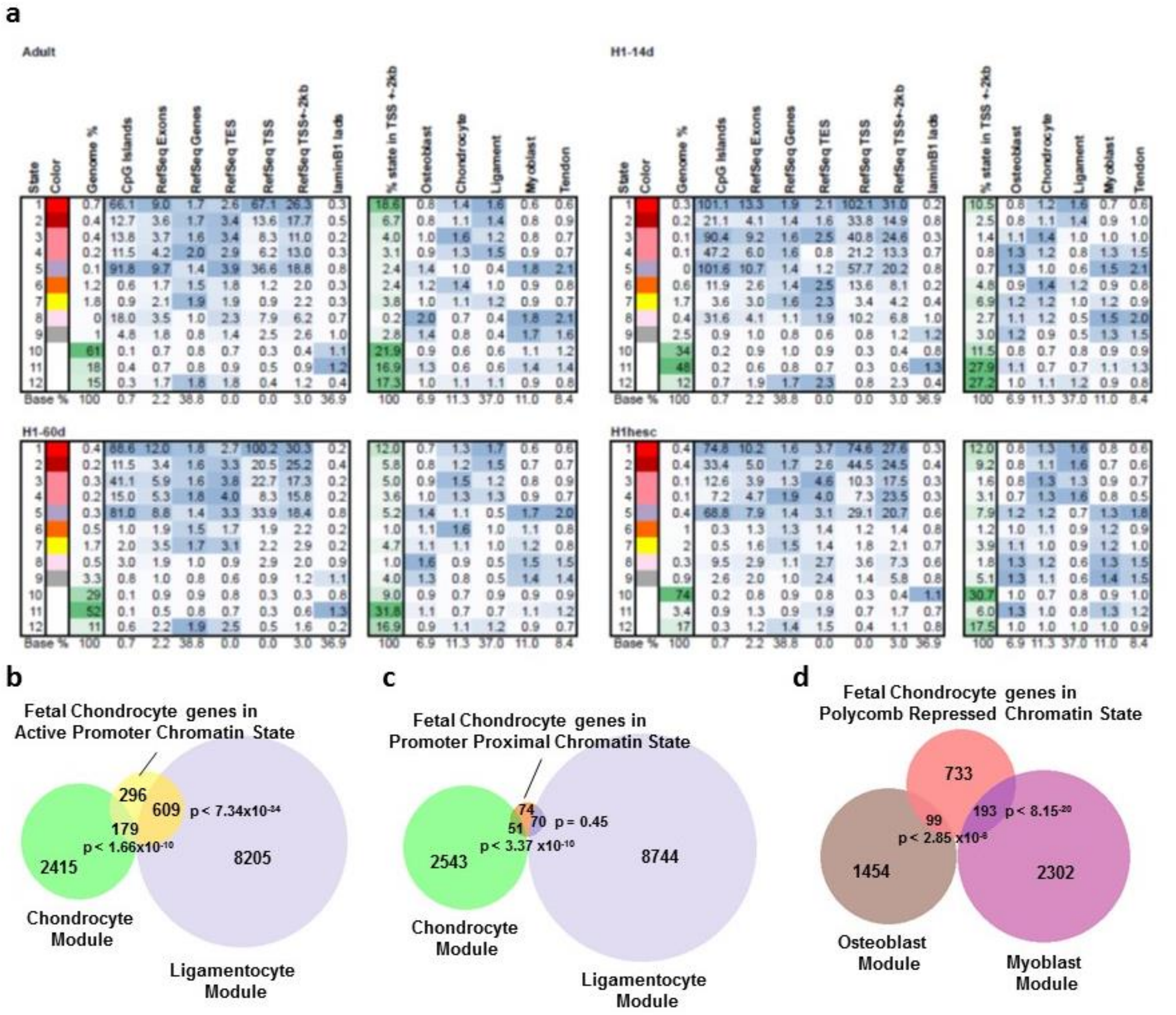

Supplementary Figure 7. Chromatin states across chondrocyte populations and gene overlaps with WGCNA gene modules. Chromatin states defined in Fig. 6. (a) Enrichments of chromatin states from Adult, H1 derived Day 14 and H1 derived Day 60 chondrocytes alongside H1 human embryonic stem cell in TSS proximal regions (defined as TSS +-2kb) of genes from different gene expression modules identified by WGCNA. The first column shows the baseline percentage occupied by each chromatin state in all annotated TSS +-2kb regions on the scale from 0 (white) to 100 (green). Subsequent columns show the enrichments for each state in each expression module based on the fraction occupied by that state in the TSS proximal regions of all genes. Each column is colored from 0 (white) to its maximum value (blue). The bottom row shows the baseline fraction of each module out of all genes. Promoters of cartilage and ligament genes are enriched for active promoter states, whereas bone, muscle and tendon genes are enriching for Polycomb-repressed, poised and low signal states. (b-d) Overlap of genes expressed in fetal chondrocytes that fall into Active Promoter, Promoter Proximal and Polycomb Repressed chromatin states (TSS +- 2kb) and WGCNA defined gene modules. (b) Overlap of Active Promoter genes with the chondrocyte and ligamentocyte modules. (c) Overlap of Promoter Proximal genes with chondrocyte and ligamentocyte modules. (d) Overlap of Polycomb Repressed genes with osteoblast and myoblast modules. N = 3-4.

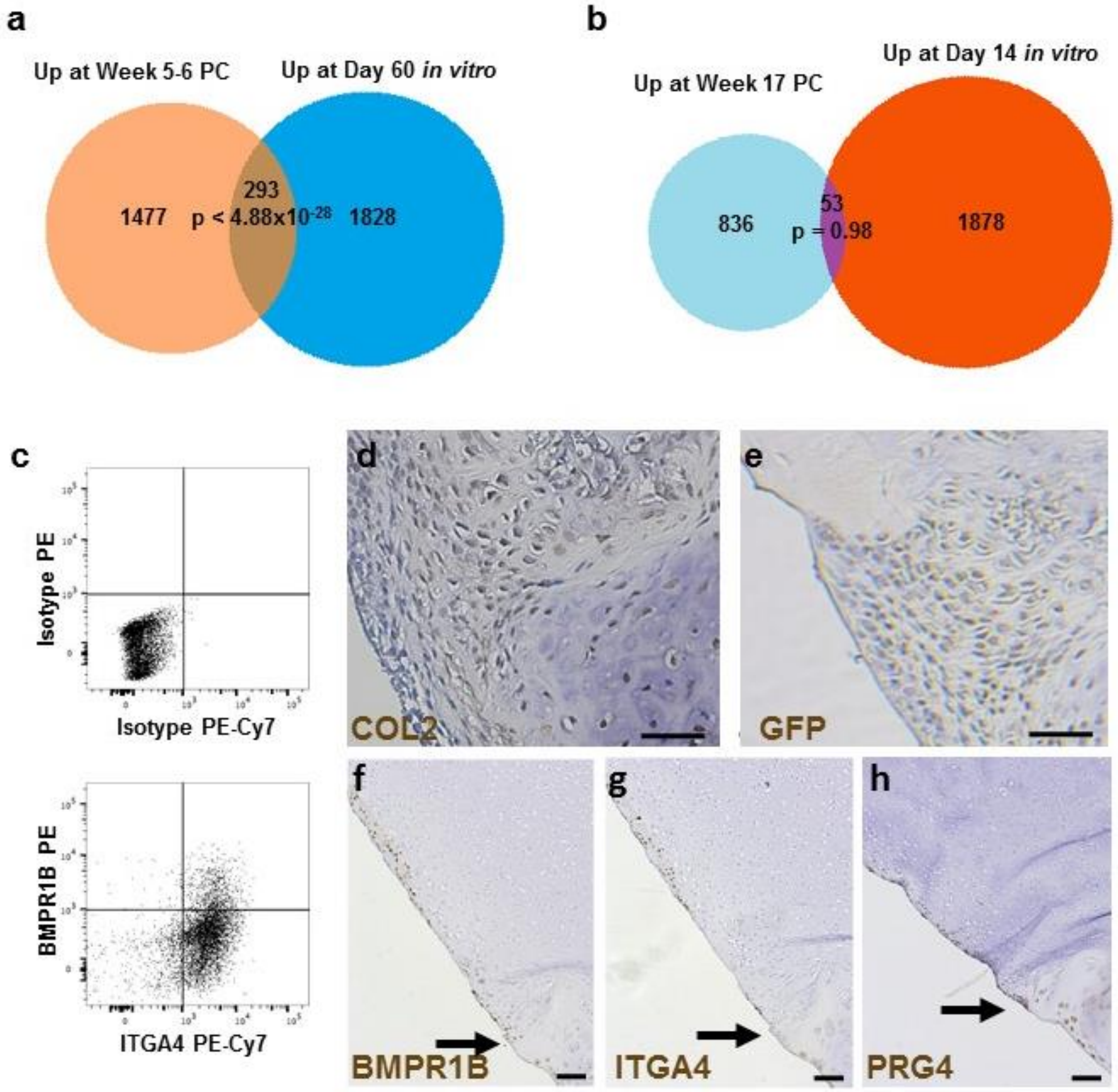

Supplementary Figure 8. Expanded analysis of human d60 PSC-derived cells before and after injection into rat articular cartilage. (a) Overlap of genes enriched in embryonic 5-6 WPC and d60 PSC-derived cartilage cells. (b) Overlap of genes enriched in fetal 17 WPC and d14 PSC-derived cartilage. Compare to Figure 7b. (c) Flow cytometric analysis of d60 chondrocytes for ITGA4 and BMPR1B. (d-h) GFP-labeled d60 chondrocytes implanted into a defect in rat knee articular cartilage. (d) Collagen 2 expression. (e) GFP protein. (f-h) Arrow denotes border of implanted d60 cells (above arrow) and endogenous rate cartilage (below arrow). Localization of BMPR1B (f), ITGA4 (g) and PRG4 (h) in superficial cells filling the defect. Lower magnification images of those in Figure 7i-k. N = 3; scale bars = 50 µm.

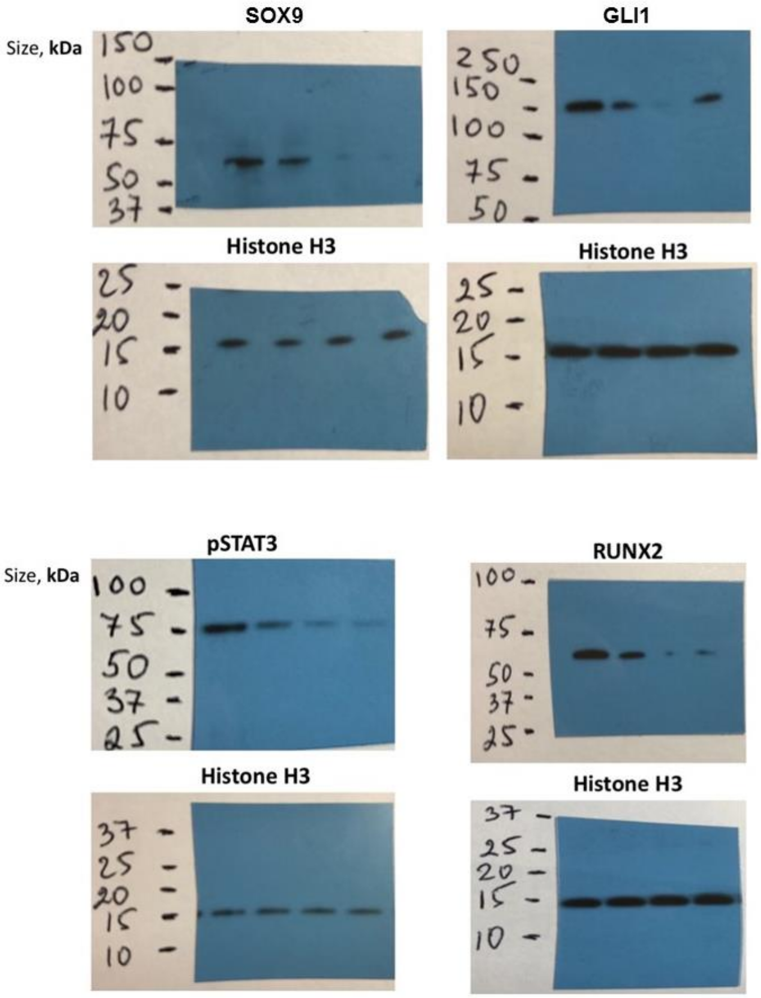

Supplementary Figure 9. Uncropped examples of Western blots.
