# Supplementary material for: Mapping molecular landmarks of human skeletal ontogeny and pluripotent stem cell-derived articular chondrocytes
Source: Nat Commun. 2018 Sep 7;9:3634. doi: 10.1038/s41467-018-05573-y (PMC6128860; doi:10.1038/s41467-018-05573-y)
Supplement: Supplementary file 2 — Description of Additional Supplementary Files [file 41467_2018_5573_MOESM2_ESM.pdf]

## Description of Additional Supplementary Files

**File Name: Supplementary Data 1**
**Description:** Genes significantly differentially expressed between 17 WPC BMPR1B- and BMPR1B+ presumptive articular chondrocytes. Positive sign indicates enrichment in BMPR1B- cells.

**File Name: Supplementary Data 2**
**Description:** Gene modules defined by WGCNA performed on fetal 17 WPC tissues.

**File Name: Supplementary Data 3**
**Description:** Expression levels (transcripts per million, TPM) for membrane proteins and transcription factors as well as and GO terms enriched in the yellow (chondrocyte) module.

**File Name: Supplementary Data 4**
**Description:** Expression levels (transcripts per million, TPM) for membrane proteins and transcription factors as well as GO terms enriched in the green (ligamentocyte) module.

**File Name: Supplementary Data 5**
**Description:** Expression levels (transcripts per million, TPM) for membrane proteins and transcription factors as well as GO terms enriched in the turquoise (myoblast) module.

**File Name: Supplementary Data 6**
**Description:** Expression levels (transcripts per million, TPM) for membrane proteins and transcription factors as well as GO terms enriched in the brown (osteoblast) module.

**File Name: Supplementary Data 7**
**Description:** Expression levels (transcripts per million, TPM) for membrane proteins and transcription factors as well as GO terms enriched in the blue (tenocyte) module.

**File Name: Supplementary Data 8**
**Description:** Transcription factor binding motifs enriched in week 17 WGCNA modules using OPPOSSUM. Z scores and Fisher scores as measures of significance are listed.

**File Name: Supplementary Data 9**
**Description:** Gene modules defined by WGCNA performed across 4 stages of human cartilage ontogeny.

**File Name: Supplementary Data 10**
**Description:** Genes significantly differentially enriched in Fetal 17 WPC vs. Adolescent chondrocytes ($p<0.01$). Negative sign indicates enrichment in fetal cells.

**File Name: Supplementary Data 11**
**Description:** Genes significantly differentially enriched in Adolescent vs. Fetal 17 WPC chondrocytes ($p<0.01$). Positive sign indicates enrichment in adolescent cells.

**File Name: Supplementary Data 12**
**Description:** Genes significantly differentially enriched in Adolescent vs. Adult chondrocytes ($p<0.01$). Positive sign indicates enrichment in adolescent cells.

**File Name: Supplementary Data 13**
**Description:** Genes significantly differentially enriched in Adult vs. Adolescent chondrocytes ($p<0.01$). Negative sign indicates enrichment in adult cells.

**File Name: Supplementary Data 14**
**Description:** Genes significantly differentially enriched in Fetal 17 WPC vs. Adult chondrocytes ($p<0.05$). Positive sign indicates enrichment in fetal cells.

**File Name: Supplementary Data 15**
**Description:** Genes significantly differentially enriched in embryonic 5-6 WPC vs. fetal 17 WPC chondrocytes ($p<0.05$). Negative sign indicates enrichment in embryonic cells.

**File Name: Supplementary Data 16**
**Description:** Transcription factor binding motifs enriched across human cartilage ontogeny using OPPOSSUM. Z scores and Fisher scores as measures of significance are listed.

**File Name: Supplementary Data 17**
**Description:** Genes significantly differentially enriched in Mouse vs. Human chondrocytes ($p<0.01$). Positive sign indicates enrichment in mouse cells.

**File Name: Supplementary Data 18**
**Description:** Genes significantly differentially enriched in Human vs. Mouse chondrocytes ($p<0.01$). Negative sign indicates enrichment in human cells.

**File Name: Supplementary Data 19**
**Description:** Genes significantly differentially expressed in d14 PSC-derived vs. d60 PSC-derived chondrocytes ($p<0.05$). Negative sign indicates enrichment in d14 cells.

**File Name: Supplementary Data 20**
**Description:** Genes significantly differentially enriched in adult pig $ITGA4^{-}BMPR1B^{-}$ articular chondrocytes vs. $CD146^{+}$ synovial pericytes ($p<0.05$). Positive sign indicates enrichment in $ITGA4^{-}BMPR1B^{-}$ cells.

**File Name: Supplementary Data 21**
**Description:** Genes significantly differentially enriched in adult pig ITGA4$^-$BMPR1B$^+$ articular chondrocytes vs. CD146$^+$ synovial pericytes ($p<0.05$). Positive sign indicates enrichment in ITGA4$^-$BMPR1B$^+$ cells.

**File Name: Supplementary Data 22**
**Description:** Genes significantly differentially enriched in adult pig ITGA4$^+$BMPR1B$^-$ articular chondrocytes vs. CD146$^+$ synovial pericytes ($p<0.05$). Positive sign indicates enrichment in ITGA4$^+$BMPR1B$^-$ cells.

**File Name: Supplementary Data 23**
**Description:** Genes significantly differentially enriched in adult pig ITGA4$^+$BMPR1B$^+$ articular chondrocytes vs. CD146$^+$ synovial pericytes ($p<0.05$). Positive sign indicates enrichment in ITGA4$^+$BMPR1B$^+$ cells.

**File Name: Supplementary Data 24**
**Description:** Genes significantly differentially enriched in adult pig ITGA4$^-$BMPR1B$^+$ vs. ITGA4$^-$BMPR1B$^-$ articular chondrocytes ($p<0.05$). Positive sign indicates enrichment in ITGA4$^-$BMPR1B$^+$ cells.

**File Name: Supplementary Data 25**
**Description:** Genes significantly differentially enriched in adult pig ITGA4$^+$BMPR1B$^-$ vs. ITGA4$^-$BMPR1B$^-$ articular chondrocytes ($p<0.05$). Positive sign indicates enrichment in ITGA4$^+$BMPR1B$^-$ cells.

**File Name: Supplementary Data 26**
**Description**: Genes significantly differentially enriched in adult pig ITGA4$^+$BMPR1B$^+$ vs. ITGA4$^-$BMPR1B$^-$ articular chondrocytes ($p<0.05$). Positive sign indicates enrichment in ITGA4$^+$BMPR1B$^-$ cells.

**File Name: Supplementary Data 27**
**Description:** Genes significantly differentially enriched in adult pig ITGA4$^+$BMPR1B$^-$vs. ITGA4$^-$BMPR1B$^+$ articular chondrocytes ($p<0.05$). Positive sign indicates enrichment in ITGA4$^+$BMPR1B$^-$ cells.

**File Name: Supplementary Data 28**
**Description:** Genes significantly differentially enriched in adult pig ITGA4$^+$BMPR1B$^+$ vs. ITGA4$^-$BMPR1B$^+$ articular chondrocytes ($p<0.05$). Positive sign indicates enrichment in ITGA4$^+$BMPR1B$^+$ cells.

**File Name: Supplementary Data 29**
**Description:** Genes significantly differentially enriched in adult pig ITGA4$^+$BMPR1B$^-$ vs. ITGA4$^+$BMPR1B$^+$ articular chondrocytes ($p<0.05$). Positive sign indicates enrichment in ITGA4$^+$BMPR1B$^-$ cells.

**File Name: Supplementary Data 30**
**Description:** Genes present in each chromatin state in fetal 17 WPC chondrocytes. Active promoter = H3K4me4, H3K27Ac; Promoter proximal = H3K4me1, H3K4me3 and H3K27Ac; Polycomb repressed = H3K27me3.

**File Name: Supplementary Data 31**
**Description:** Genes present in each chromatin state in adult articular chondrocytes. Active promoter = H3K4me4, H3K27Ac; Promoter proximal = H3K4me1, H3K4me3 and H3K27Ac; Polycomb repressed = H3K27me3.

**File Name: Supplementary Data 32**
**Description:** Genes present in each chromatin state in d14 PSC-derived chondrocytes. Active promoter = H3K4me4, H3K27Ac; Promoter proximal = H3K4me1, H3K4me3 and H3K27Ac; Polycomb repressed = H3K27me3.

**File Name: Supplementary Data 33**
**Description:** Genes present in each chromatin state in d60 PSC-derived chondrocytes. Active promoter = H3K4me4, H3K27Ac; Promoter proximal = H3K4me1, H3K4me3 and H3K27Ac; Polycomb repressed = H3K27me3.

**File Name: Supplementary Data 34**
**Description:** Genes, and GO terms, enriched commonly in fetal 17 WPC chondrocytes vs. embryonic 5-6 WPC pre-chondrocytes and d60 vs. d14 PSC-derived chondrocytes.

**File Name: Supplementary Data 35**
**Description:** Genes, and GO terms, enriched commonly in embryonic 5-6 WPC pre-chondrocytes vs. fetal 17 WPC chondrocytes and d14 vs. d60 PSC-derived chondrocytes.

**File Name: Supplementary Data 36**
**Description:** Metrics for samples sequenced including total reads and mapped reads.
